# Plasma Adiponectin Levels and Risk of Heart Failure, Atrial Fibrillation, Aortic Valve Stenosis, and Myocardial Infarction: Large scale observational and Mendelian Randomization evidence

**DOI:** 10.1093/cvr/cvad162

**Published:** 2023-10-28

**Authors:** Maria Booth Nielsen, Yunus Çolak, Marianne Benn, Amy Mason, Stephen Burgess, Børge Grønne Nordestgaard

**Affiliations:** 1 Department of Clinical Biochemistry, Copenhagen University Hospital - Herlev and Gentofte, Copenhagen, Denmark; 2 The Copenhagen General Population Study, Copenhagen University Hospital - Herlev and Gentofte, Copenhagen, Denmark; 3 Department of Clinical Medicine, Faculty of Health and Medical Sciences, University of Copenhagen, Copenhagen, Denmark; 4 Department of Respiratory Medicine, Copenhagen University Hospital - Herlev and Gentofte, Copenhagen, Denmark; 5 Department of Clinical Biochemistry, Copenhagen University Hospital - Rigshospitalet, Copenhagen, Denmark; 6 Medical Research Council Biostatistics Unit, University of Cambridge, Cambridge, United Kingdom; 7 British Heart Foundation Cardiovascular Epidemiology Unit, Department of Public Health and Primary Care, University of Cambridge, Cambridge, United Kingdom; 8 Heart and Lung Research Institute, University of Cambridge, Cambridge, United Kingdom

**Keywords:** Adiponectin, Heart Failure, Atrial Fibrillation, Aortic Valve Stenosis, Myocardial Infarction, Genetic Polymorphisms

## Abstract

**Aims:**

Adiponectin may play an important protective role in heart failure and associated cardiovascular diseases. We hypothesized that plasma adiponectin is associated observationally and causally, genetically with risk of heart failure, atrial fibrillation, aortic valve stenosis, and myocardial infarction.

**Methods and results:**

In the Copenhagen General Population Study, we examined 30,045 individuals with plasma adiponectin measurements observationally and 96,903 individuals genetically in one-sample Mendelian randomization analyses using five genetic variants explaining 3% of the variation in plasma adiponectin. In the HERMES, UK Biobank, The Nord-Trøndelag Health Study(HUNT), deCODE, the Michigan Genomics Initiative(MGI), DiscovEHR, and the AFGen consortia, we performed two-sample Mendelian randomization analyses in up to 1,030,836 individuals using 12 genetic variants explaining 14% of the variation in plasma adiponectin.

In observational analyses modelled linearly, a 1 unit log-transformed higher plasma adiponectin was associated with a hazard ratio of 1.51(95% confidence interval:1.37–1.66) for heart failure, 1.63(1.50–1.78) for atrial fibrillation, 1.21(1.03–1.41) for aortic valve stenosis, and 1.03(0.93–1.14) for myocardial infarction; levels above the median were also associated with increased risk of myocardial infarction, and non-linear U-shaped associations were more apparent for heart failure, aortic valve stenosis, and myocardial infarction in less-adjusted models. Corresponding genetic, causal risk ratios were 0.92(0.65–1.29), 0.87(0.68–1.12), 1.55(0.87–2.76), and 0.93(0.67–1.30) in one-sample Mendelian randomization analyses, and no significant associations were seen for non-linear one-sample Mendelian randomization analyses; corresponding causal risk ratios were 0.99(0.89–1.09), 1.00(0.92–1.08), 1.01(0.79–1.28), and 0.99(0.86–1.13) in two-sample Mendelian randomization analyses, respectively.

**Conclusion:**

Observationally, elevated plasma adiponectin was associated with increased risk of heart failure, atrial fibrillation, aortic valve stenosis, and myocardial infarction. However, genetic evidence did not support causality for these associations.

## 1 Introduction

The adipocyte-secreted protein-hormone adiponectin potentially plays an important role in heart failure. Adiponectin exerts insulin-sensitizing, anti-atherogenic, and anti-inflammatory properties in preclinical studies^1, 2^, supportive of a cardiovascular protective role^3^. In human observational studies, however, the picture is less clear. While adiponectin seems to be inversely associated with cardiovascular risk factors such as body mass index(BMI), type 2 diabetes, and abdominal fat accumulation, in contrast to other adipokines^4–6^, its association with heart failure and associated cardiovascular diseases seems to be inconsistent^7–12^. It is therefore unclear whether adiponectin is a causal risk factor for heart failure. Unravelling this unclarity is pivotal in understanding etiological mechanisms, potentially discovering novel drug targets, and designing lifestyle-interventions to prevent and treat heart failure.

Mendelian randomization is an approach to investigate causal relationships by taking advantage of natural randomization of genetic variants and utilizing them as instrumental variables to estimate the potential effect of an exposure on an outcome. At conception genes are already present and alleles randomly distributed; thus, Mendelian randomization is less vulnerable to reverse causation and genetic variants are generally not associated with potential confounders^13–15^. Genome-wide associations studies have identified genetic variants associated with plasma adiponectin^16–18^, making it possible to investigate adiponectin as a risk factor.

**Figure 1:**
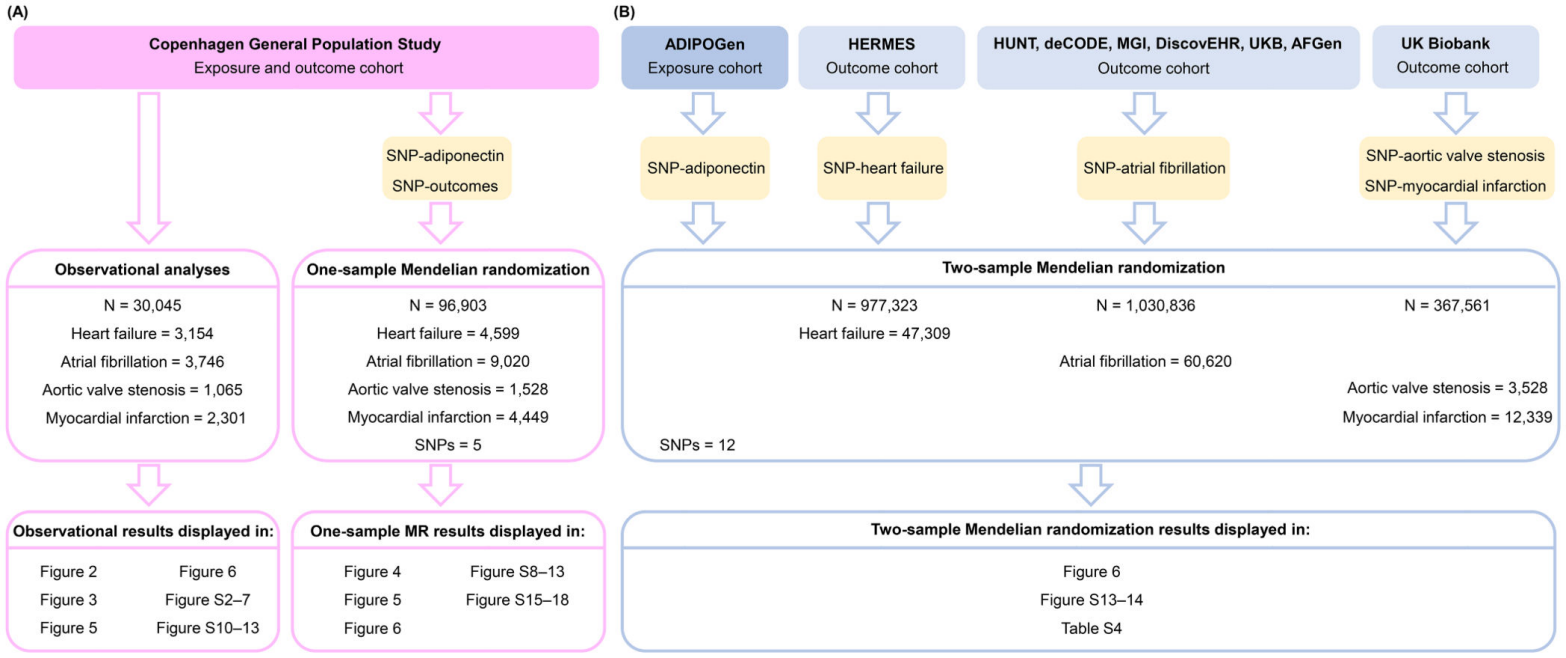
Flowchart for observational and genetic Mendelian randomization analyses. (A) In observational and genetic one-sample Mendelian randomization analyses, the Copenhagen General Population Study was used as exposure and outcome cohort(pink). (B) In genetic two-sample Mendelian randomization analyses, ADIPOGen was used as the exposure cohort and HERMES, UK Biobank(UKB), The Nord-Trøndelag Health Study(HUNT), deCODE, the Michigan Genomics Initiative(MGI), DiscovEHR, and the AFGen consortia as the outcome cohorts(blue). The supplementary material contains summarized descriptions of the included cohorts. SNPs=single nucleotide polymorphisms. MR=Mendelian randomization.

We hypothesized that plasma adiponectin is associated observationally and causally, genetically with risk of heart failure, atrial fibrillation, aortic valve stenosis, and myocardial infarction. We used the Copenhagen General Population Study with information on 30,045 and 96,903 individuals in observational and genetic one-sample Mendelian randomization analyses(Figure 1, **Part A**). In addition, we used information on 361,194–1,030,836 individuals from the ADIPOGen, HERMES, UK Biobank(UKB), The Nord-Trøndelag Health Study(HUNT), deCODE, the Michigan Genomics Initiative(MGI), DiscovEHR, and the AFGen consortia in genetic two-sample Mendelian randomization analyses(Figure 1, **Part B**) to increase statistical power and validate findings.

## 2 Methods

### 2.1 Observational and genetic one-sample Mendelian randomization study design

We included individuals aged 20–100 years from the Copenhagen General Population Study(CGPS), a population-based cohort from 2003 with ongoing enrolment. Individuals were randomly invited from the national Danish Civil Registration system to reflect the adult white population of Danish descent; being of Danish descent is defined in the national Danish Civil Registration system as a person jointly with both parents all being born in Denmark and with Danish citizenship, while the remainder of individuals in the population are registered either as immigrants or descendants of immigrants, where the latter two groups are not included in the CGPS. All participants completed a questionnaire, had a physical examination, and had blood drawn for biochemical and genetical testing^5, 6, 19^. The study was approved by a Danish ethical committee(approval number:H-KF-01-144/01) and conducted according to the Declaration of Helsinki. All participants provided written informed consent.

Plasma adiponectin was measured using a latex-enhanced turbidimetric immunoassay on a Cobas® autoanalyzer(Roche) and measurements were conducted blind to information on genetic variants and outcomes^5, 6, 19^. Measurement of plasma adiponectin was available for 30,045 individuals.

We genotyped five genetic variants associated with plasma adiponectin that had the lowest P-values and largest effect sizes according to genome-wide associations studies^16–18^. Importantly, we selected these biologically relevant genetic variants in the *ADIPOQ* and *CDH13* loci encoding respectively for plasma adiponectin and T-cadherin, a receptor recognizing adiponectin(Table S1, **pink column**). While a TaqMan-like method by LCG Genomics(Teddington) was used to genotype for rs2062632, rs266717, and rs6810075, the ABI PRISM 7900HT Sequence Detection System(Applied Biosystems) was used with TaqMan assays to genotype for rs17366568 and rs2925979. Genotyping was conducted blind to information on plasma adiponectin and outcomes. There was no indication of linkage disequilibrium between pairwise combinations of the four genetic variants in the *ADIPOQ* locus(all R^2^ <0.24; Table S2)^5, 6, 19, 20^. We had information on all five genetic variants for 96,903 individuals^6, 19^.

Clinical outcomes included heart failure(International Classification of Diseases [ICD]-8:427.09–427.11 and ICD-10:I50.0, I50.1, and I50.9), atrial fibrillation(ICD-8:427.93–427.93 and ICD-10:I48.0–I48.9), aortic valve stenosis(ICD-8:424.10, 424.12, 424.18, 424.19 and ICD-10:I35 and I35.2), and myocardial infarction(ICD-8:410 and ICD-10:I21–I22) collected from the national Danish Patient Registry, which records all physician diagnosed public and private hospital contacts in Denmark, from 1977 through 2018. Diagnoses from the national Danish Patient Registry has previously been evaluated with positive predictive values(PPV) ≥90% for cardiovascular outcomes included in the present study, indicating an overall high validity^21, 22^. While the PPV for aortic valve stenosis was not evaluated individually but as a part of aortic valve disorders,^21^ another study found a PPV of up to 80% for the diagnosis of aortic valve disorders still suggesting a high validity^23^.

Covariates used for adjustment and stratification in observational analyses (and for stratification in genetic analyses) included hypertension, diabetes, use of lipid-lowering drugs, smoking status, socioeconomic status, physical activity, body mass index, waist circumference, non-high-density lipoprotein cholesterol, and plasma high-sensitive C-reactive protein. Hypertension was systolic blood pressure ≥140 mmHg, diastolic blood pressure ≥90 mmHg, systolic blood pressure ≥130 mmHg and diabetes, diastolic blood pressure ≥85 mmHg and diabetes, and/or use of antihypertensive medication. Baseline diabetes was based on self-report, non-fasting plasma glucose >11 mmol/L(198 mg/dL), use of antidiabetic medication, and/or previous inpatient/outpatient hospital contact identified through the national Danish Patient Registry(ICD-8:249–250 and ICD-10:E10–E14). Use of lipid-lowering drugs was self-reported. Body mass index(BMI) was based on measured weight divided by measured height squared(kg/m^2^). Waist circumference(cm) was also measured. Smoking status was categorized as current smoker or non-smoker. Alcohol consumption was reported in units per week(1 unit=12 g). Socioeconomic status was based on education and annual household income. Degree of leisure-time physical activity was self-reported. Plasma C-reactive protein(mg/L), high-density lipoprotein(HDL) cholesterol(mmol/L), and plasma total cholesterol(mmol/L) were measured using standard hospital assays. Non-HDL cholesterol(mmol/L) was calculated by subtracting HDL cholesterol from plasma total cholesterol.

### 2.2 Genetic two-sample Mendelian randomization study design

From the ADIPOGen consortium^18^ we identified genetic variants associated with plasma adiponectin (=SNP-plasma adiponectin) that reached genome-wide significance threshold and variants in linkage disequilibrium were removed. Thereafter, we identified the same genetic variants in the clinical outcome cohorts: i) (=SNP-heart failure) from the HERMES^24^, ii) (=SNP-atrial fibrillation) from the HUNT, deCODE, MGI, DiscovEHR, UKB, and the AFGen consortia^25^, iii) (=SNP-aortic valve stenosis) from the UKB^26^, and (iv) (=SNP-myocardial infarction) from the UKB^26^. We combined the genetic information on exposure and clinical outcomes in two-sample Mendelian randomization analyses to estimate the effect of plasma adiponectin on outcome risk.

We used the MR-base software and MR_Practicals R package with information on linkage structure in 3,775 genomes from the 1000 Genomes Project, as done previously^19, 27^. Palindromic genetic variants (rs7964945 and rs2980879) were removed due to difficulty harmonizing the effect alleles in the exposure sample with the corresponding alleles in all outcome samples^27^.

From the ADIPOGen consortium, we included 12 genetic variants associated with plasma adiponectin; in sensitivity analyses we also studied selectively those variants in the *ADIPOQ* locus similar to that done in the one-sample Mendelian randomization analyses(Table S1, **blue column**). In the outcome samples, we harmonized and tested the genetic variants against the outcomes i) heart failure(ID:ebi-a-GCST009541)^24^, ii) atrial fibrillation(ebi-a-GCST006414)^25^, iii) aortic valve stenosis(UKB)^26^, and iv) myocardial infarction(UKB)^26, 27^.

### 2.3 Statistical analyses

We used STATA/SE 15.1 and R 3.6.1 for Windows.

#### 2.3.1 Observational analyses

Observational association of plasma adiponectin with clinical outcomes was investigated using multivariable adjusted Cox proportional hazards regression with age as timescale(=age adjusted) and left truncation(=delayed entry) at study entry(Figure S1, **Step 1**). We tested the Cox proportional hazard model assumptions with no major violations observed. We used both untransformed plasma adiponectin in μg/mL and natural log-transformed plasma adiponectin, the latter to compare observational estimates with one- and two-sample Mendelian randomization estimates. First, we investigated the association as a dose-response relationship using restricted cubic splines with the median value of plasma adiponectin as the reference. Second, we investigated such associations using quartiles of plasma adiponectin. The quartiles were based on median values of plasma adiponectin within each quartile and graphically displayed using kernel-weighted local polynomial smoothing and geometric means with 95% confidence intervals(CIs). Third, we investigated the association per 1 unit higher plasma adiponectin using both untransformed and natural log-transformed plasma adiponectin. Observational analyses were adjusted for potential confounders age(as timescale), sex, hypertension, diabetes, use of lipid-lowering drugs, smoking status, socioeconomic status, physical activity, BMI, waist circumference, non-HDL cholesterol, and plasma high-sensitive C-reactive protein. Some of the participants lacked information on some potential confounders(missing covariates were 1.4%). Therefore, we did multiple imputation using chained equations to fill in missing values; however, results were similar without imputation. To investigate for potential effect modification(=interaction) in observational analyses, risk of clinical outcomes was also investigated jointly with plasma adiponectin and other relevant risk factors, including sex, age, BMI, waist circumference, hypertension, and prevalent cardiovascular disease using a likelihood ratio test in models with and without two-factor interaction terms. Furthermore, reverse causation was investigated by comparing individuals in the 4th quartile(highest plasma adiponectin) with those in the 1st quartile(lowest plasma adiponectin) and excluding individuals with less than one to four years of follow-up from the analyses.

#### 2.3.2 Genetic one-sample Mendelian randomization analyses

Deviation of the genetic variants from Hardy-Weinberg equilibrium was investigated using Pearson’s chi-squared test. To assess strength of the genetic variants as instrumental variables, we calculated the F-value and the variation in plasma adiponectin explained by the genetic variants using a linear regression. Associations between the genetic adiponectin score and potential confounders were investigated using linear and logistic regressions. Association of the internally weighted genetic adiponectin score with plasma adiponectin was investigated using linear regression and graphically displayed using geometric means(Figure S1, **Step 2**). Associations of the internally weighted genetic adiponectin score with clinical outcomes were investigated using multivariable logistic regression(Figure S1, **Step 3**). Instrumental variable estimates of causal risk ratios were calculated using the Wald-type estimator and internally weighted genetic adiponectin score to estimate the influence of genetically determined plasma adiponectin with risk of heart failure, atrial fibrillation, aortic valve stenosis, and myocardial infarction(Figure S1, **Step 4**). To assess potential bias towards the observational estimate with the use of an internally weighted genetic score^28^, we additionally used an unweighted and externally weighted genetic adiponectin score in sensitivity analyses. The external weighted score was created using coefficients from the ADIPOGen consortia^18^. All one-sample genetic analyses were adjusted for age and sex. To assess potential pleiotropy in one-sample Mendelian randomization and to compare with results from two-sample Mendelian randomization, different analytical methods were used: inverse-variance weighted(IVW), Mendelian randomization-Egger(MR-Egger), weighted median estimates, and weighted mode regressions. For this purpose, we also natural log-transformed plasma adiponectin in the Copenhagen General Population Study. Furthermore, to investigate for potential effect modification(=interaction) in genetic one-sample Mendelian randomization analyses, risk of clinical outcomes was also investigated jointly with plasma adiponectin and other relevant risk factors, including sex, age, BMI, waist circumference, hypertension, and prevalent cardiovascular disease using a likelihood ratio test in models with and without two-factor interaction terms. Lastly, to investigate the shape of a potential causal relationship, we applied a non-linear Mendelian randomization approach^29, 30^ and graphically displayed the results using fractional polynomial and piecewise linear methods from the SUMnlmr R package^31^. For this purpose, we divided the population into ten strata via the novel doubly-ranked stratification method^29–31^ on genetic adiponectin score and natural log-transformed plasma adiponectin.

#### 2.3.3 Genetic two-sample Mendelian randomization analyses

In genetic two-sample Mendelian randomization analyses, we extracted summary data on plasma adiponectin from the MR Base GWAS catalogue, pruned for linkage disequilibrium between genetic variants and extracted summary data on heart failure and atrial fibrillation^27^. Updated summary data on aortic valve stenosis and myocardial infarction from the UKB were uploaded manually. We harmonized the exposure and outcome datasets. Finally, we did Mendelian randomization instrumental variable analyses using IVW, MR-Egger, weighted median estimates, and weighted mode regressions. For this purpose, we used the MR_Practicals R package including MRInstruments and TwoSampleMR^27, 32^. To assess instrument strength, we calculated the F-value as $F=\frac{N-K-1}{K}\cdot \frac{R^{2}}{1-R^{2}}$^33^.

We used an online power calculator to determine the causal effect we can detect with 80% power in one- and two-sample Mendelian randomization analyses(Table S3)^34^.

## 3 Results

### 3.1 Observational results in the CGPS

Baseline characteristics in the CGPS is summarised in Table 1. Plasma adiponectin was associated with all potential confounders(Table 1).

**Figure 2:**
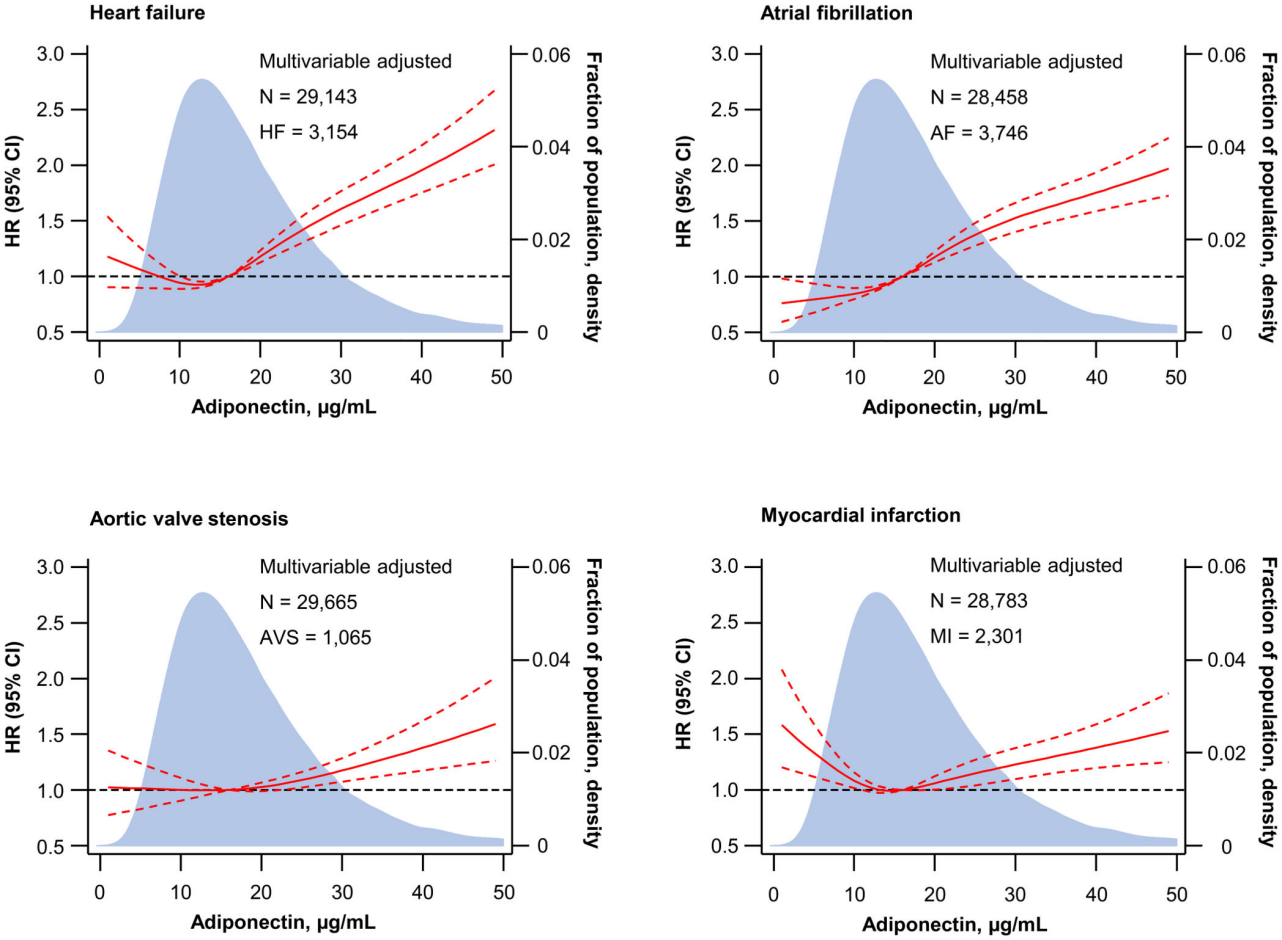
Observational association of plasma adiponectin with heart failure(HF), atrial fibrillation(AF), aortic valve stenosis(AVS), and myocardial infarction(MI) in the Copenhagen General Population Study. Hazard ratios(HR) are indicated with a solid red line and 95% confidence interval(CI) with dashed red lines. Median concentration of plasma adiponectin(16 μg/mL) was used as reference with a hazard ratio of 1.0 indicated with horizontal dashed black line. Individuals in the upper 1st percentile for plasma adiponectin(plasma adiponectin ≥50 μg/mL) were included in the analyses but excluded from the graphs for visual purposes. Analyses were multivariable adjusted for age(as timescale), sex, hypertension, diabetes, use of lipid-lowering drugs, smoking status, socioeconomic status, physical activity, body mass index, waist circumference, non-high-density lipoprotein cholesterol, and plasma high-sensitive C-reactive protein. Fraction of the population indicated with light blue. N=number of individuals.

**Figure 3:**
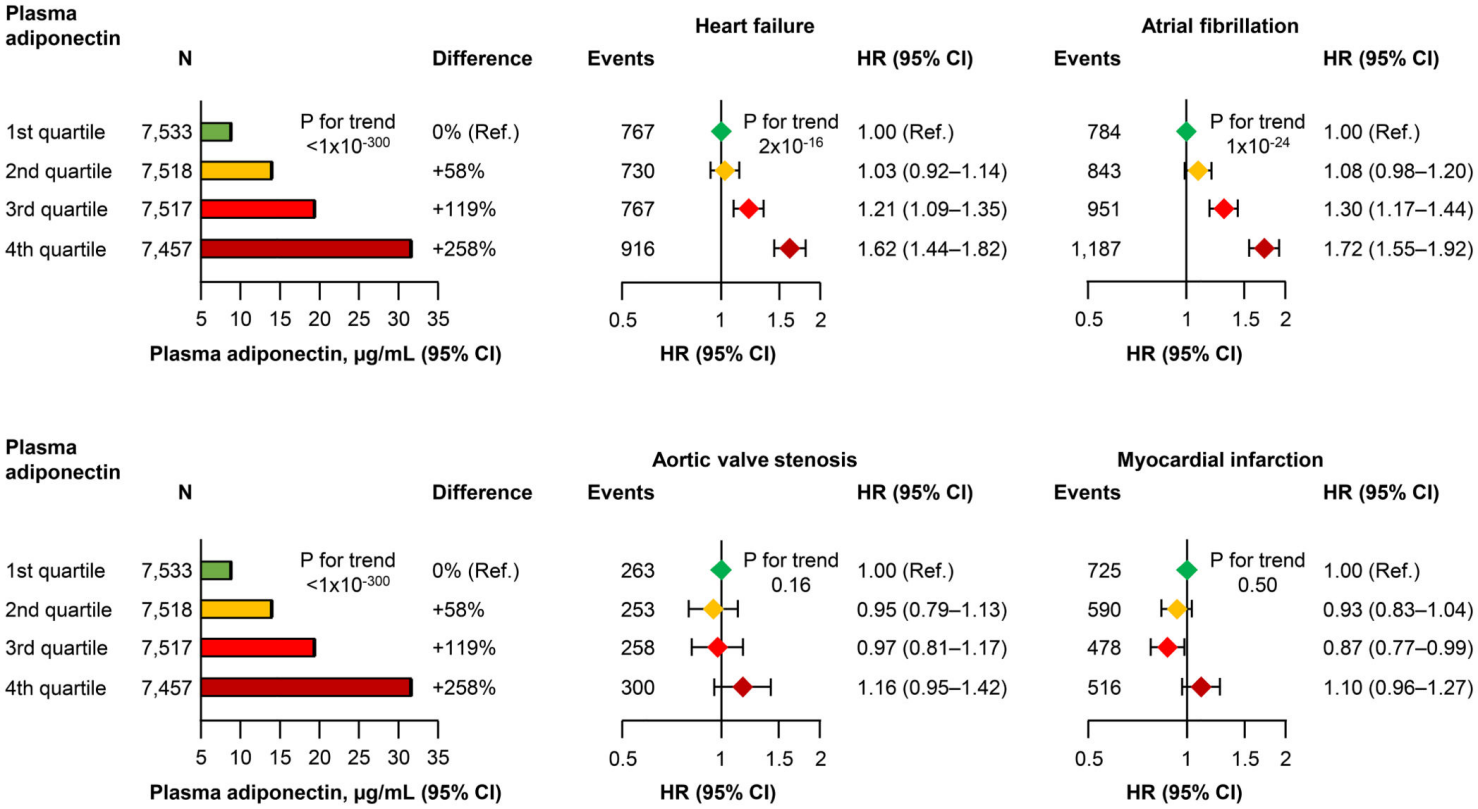
Observational association of plasma adiponectin with heart failure, atrial fibrillation, aortic valve stenosis, and myocardial infarction in the Copenhagen General Population Study. Geometric mean with 95% confidence interval(CI) for plasma adiponectin is indicated with bars and whiskers. Hazard ratios(HR) with 95% CI for heart failure, atrial fibrillation, aortic valve stenosis, and myocardial infarction are indicated with diamonds and whiskers. Analyses were multivariable adjusted for age(as timescale), sex, hypertension, diabetes, use of lipid-lowering drugs, smoking status, socioeconomic status, physical activity, body mass index, waist circumference, non-high-density lipoprotein cholesterol, and plasma high-sensitive C-reactive protein. N=number of individuals.

Elevated plasma adiponectin was associated with higher hazard ratios for heart failure, atrial fibrillation, aortic valve stenosis, and myocardial infarction after multivariable adjustment in restricted cubic splines(Figure 2). Divided into quartiles, elevated plasma adiponectin was also associated with higher hazard ratios for heart failure and atrial fibrillation but not with aortic valve stenosis or myocardial infarction(Figure 3); with a more U-shaped association for heart failure, aortic valve stenosis, and myocardial infarction(Figure 2 and 3). Results were similar in a less adjusted model by excluding factors that have previously been shown to eliminate the U-shaped association at lower plasma adiponectin concentrations^35, 36^, that is, diabetes, use of lipid-lowering drugs, non-HDL cholesterol, and plasma high-sensitive C-reactive protein(compare Figures 2–3 with Figures S2–S3). Likewise, results were similar when excluding individuals with prevalent cardiovascular disease in the extensively adjusted model and in the less adjusted model(compare Figure 2, Figure S4 and S5); however, with a more U-shaped association for heart failure and myocardial infarction. Likewise, results were similar, in a model without adjustment for body mass index and waist circumference(compare Figure 2 with Figure S6); however, the association attenuated, suggesting that obesity measures affect the risk of disease, as previously shown^37–40^. Furthermore, results were similar when smoking status was categorized as never, former, or current smoker and cumulative tobacco consumption(pack years) was included in the model(data not shown). Finally, there was no evidence of reverse causation(Figure S7).

### 3.2 Genetic results in the CGPS

There was no evidence that the genetic adiponectin score was associated with any potential confounders(all P-values ≥0.05 after taking multiple testing into account)(Table 1). There was no indication of deviation from Hardy-Weinberg equilibrium(all P-values ≥0.05).

**Figure 4:**
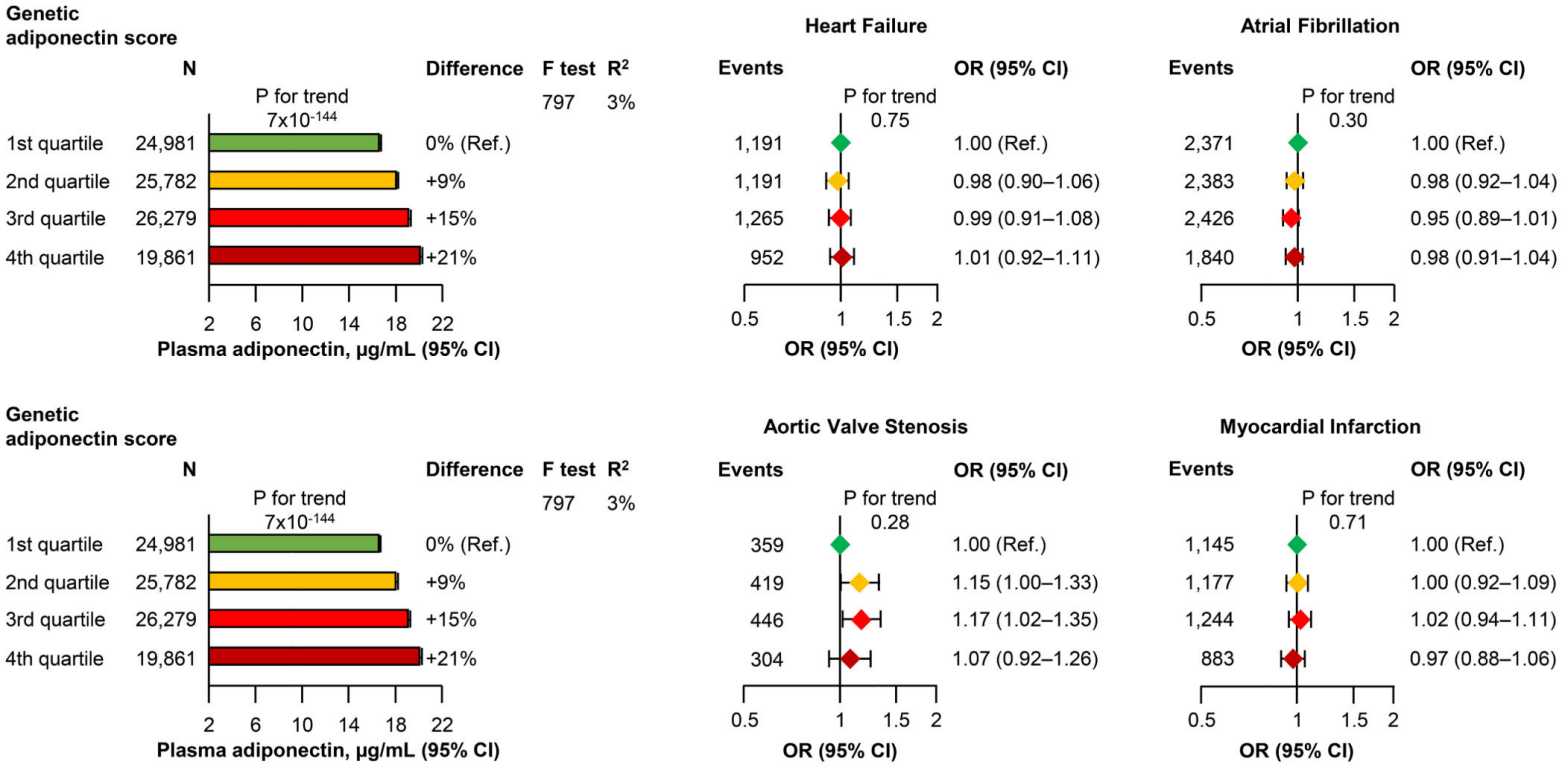
Genetic association of plasma adiponectin with heart failure, atrial fibrillation, aortic valve stenosis, and myocardial infarction in the Copenhagen General Population Study. Geometric mean with 95% confidence interval(CI) for plasma adiponectin is indicated with bars and whiskers. Odds ratio(OR) with 95% CI for heart failure atrial fibrillation, aortic valve stenosis, and myocardial infarction are indicated with diamonds and whiskers. Analyses for risk of disease were adjusted for age and sex. The genetic adiponectin score explained 3%of the variation in plasma adiponectin(R^2^) with an F-value of 797. N=number of individuals.

The internally weighted genetic adiponectin score explained 3% of the variation in plasma adiponectin with an F-value of 797. The internally weighted genetic adiponectin score in quartiles was associated with stepwise higher plasma adiponectin(Figure 4, **left part**). However, there was no association between the internally weighted genetic adiponectin score in quartiles and odds ratios for heart failure, atrial fibrillation, aortic valve stenosis, or myocardial infarction(P for trend ≥0.05)(Figure 4, **right part**). Results were similar using an unweighted and externally weighted genetic adiponectin score(compare Figure 4 with Figures S8–9).

### 3.3 Observational and genetic one-sample Mendelian randomization results in the CGPS

**Figure 5:**
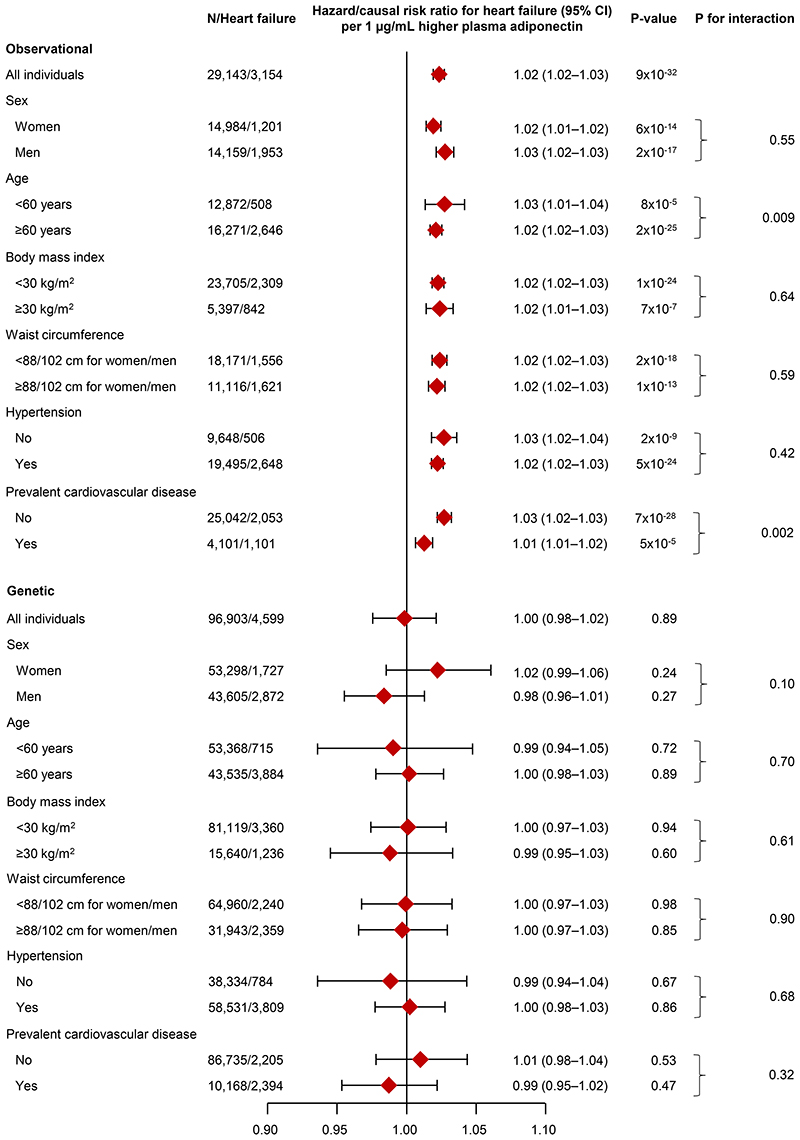
Observational and genetic one-sample Mendelian randomization of plasma adiponectin with heart failure in clinical subgroups in the Copenhagen General Population Study. Observational analyses were multivariable adjusted for age, sex, hypertension, diabetes, use of lipid-lowering drugs, smoking status, socioeconomic status, physical activity, body mass index, waist circumference, non-high-density lipoprotein cholesterol, and plasma high-sensitive C-reactive protein. Genetic results are from Wald-type estimator and analyses were adjusted for age and sex. N=number of individuals. Corresponding data for atrial fibrillation, aortic valve stenosis, and myocardial infarction is shown in Figures S10–12.

In genetic one-sample Mendelian randomization analyses using the Wald-type estimator, a 1 μg/mL higher plasma adiponectin was associated with a causal risk ratio of 1.00(95% CI:0.98–1.02) for heart failure(Figure 5, **lower part**), 0.99(0.98–1.01) for atrial fibrillation(Figure S10, **lower part**), 1.03(0.99–1.07) for aortic valve stenosis(Figure S11, **lower part**), and 0.99(0.97–1.02) for myocardial infarction(Figure S12, **lower part**). Results were similar when using an unweighted and externally weighted genetic adiponectin score(data not shown). In contrast, in observational analyses a 1 μg/mL higher plasma adiponectin was associated with a hazard ratio of 1.02(95% CI:1.02–1.03) for heart failure(Figure 5, **upper part**), 1.02(1.02–1.03) for atrial fibrillation(Figure S10, **upper part**), 1.01(1.00–1.02) for aortic valve stenosis(Figure S11, **upper part**), and 1.01(1.00–1.01) for myocardial infarction(Figure S12, **upper part**). Overall, results were similar for women and men separately, for individuals < and ≥60 years, for individuals with BMI < and ≥30 kg/m^2^, for individuals with waist circumference < and ≥88/102 cm(women/men), for individuals with or without hypertension, and for individuals with or without prevalent cardiovascular disease(P-values for interaction ≥0.05); however, in observational analyses, individuals <60 years and individuals without prevalent cardiovascular disease had a slightly higher hazard ratio for heart failure(P-value for interaction=0.009 and 0.002 respectively), while men had a slightly higher hazard ratio for atrial fibrillation(P-value for interaction=0.002)(Figure 5 and Figures S10–12, **upper parts**).

Comparison of results from observational and genetic one-sample Mendelian randomization with two-sample Mendelian randomization using natural log-transformed plasma adiponectin and IVW, MR-Egger, weighted median, and weighted mode is described below.

### 3.4 Genetic two-sample Mendelian randomization and comparison with observational and genetic one-sample Mendelian randomization results

The 12 genetic variants used in two-sample Mendelian randomization explained 14% of the variation in plasma adiponectin with an F-value of 398.

**Figure 6:**
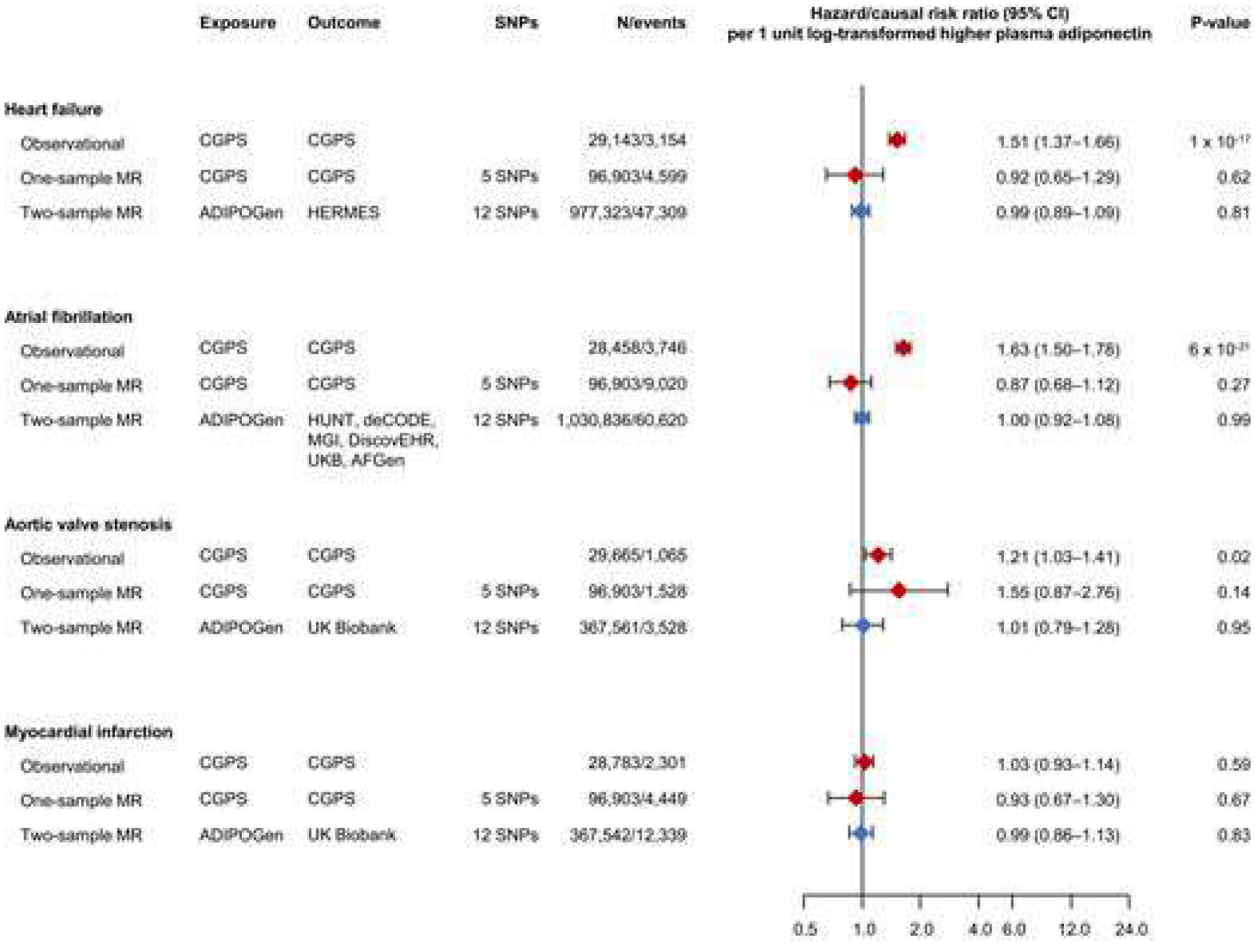
Observational and genetic one- and two sample Mendelian randomization of plasma adiponectin with heart failure, atrial fibrillation, aortic valve stenosis, and myocardial infarction. Observational analyses in the Copenhagen General Population Study(CGPS) were multivariable adjusted for age, sex, hypertension, diabetes, use of lipid-lowering drugs, smoking status, socioeconomic status, physical activity, body mass index, waist circumference, non-high-density lipoprotein cholesterol, and plasma high-sensitive C-reactive protein. The causal risk ratio with 95% confidence interval(CI) from one-sample Mendelian randomization(MR) analyses indicated with red diamonds and whiskers was based on the CGPS. The causal risk ratio with 95% CI from two-sample Mendelian randomization analyses indicated with blue diamonds and whiskers was based on HERMES in heart failure; HUNT, deCODE, MGI, DiscovEHR, UKB, and AFGen in atrial fibrillation; and UKB in aortic valve stenosis and myocardial infarction. Genetic information on plasma adiponectin for the two-sample Mendelian randomization were obtained from ADIPOGen. Genetic results are from inverse variance weighted analyses. Results from MR-Egger, weighted median, and weighted mode analyses are shown in Figure S13. SNPs=single nucleotide polymorphisms. N=number of individuals.

In observational analyses(the same study as shown in Figures 2, 3, and 5), a 1 unit log-transformed higher plasma adiponectin was associated with a hazard ratio of 1.51(95% CI:1.37–1.66) for heart failure, 1.63(1.50–1.78) for atrial fibrillation, 1.21(1.03–1.41) for aortic valve stenosis, and 1.03(0.93–1.14) for myocardial infarction(Figure 6). Corresponding genetic, causal risk ratios using IVW analysis were 0.92(0.65–1.29), 0.87(0.68–1.12), 1.55(0.87–2.76), and 0.93(0.67–1.30) in one-sample Mendelian randomization analyses, while corresponding causal risk ratios were 0.99(0.89–1.09), 1.00(0.92–1.08), 1.01(0.79–1.28), and 0.99(0.86–1.13) in two-sample Mendelian randomization analyses, respectively(Figure 6).

When using MR-Egger, weighted median, and weighted mode for heart failure, atrial fibrillation, and myocardial infarction results were similar; however, for aortic valve stenosis in one-sample Mendelian randomization, a 1 unit log-transformed higher plasma adiponectin was associated with a causal risk ratio of 5.66(1.36–23.57) in MR-Egger and 2.73(1.15–6.52) in weighted mode(compare Figure S13 with Figure 6); this apparent positive association was most likely due to chance finding since the main one-sample and additional two-sample Mendelian randomization analyses did not concur.

Results for genetic two-sample Mendelian randomization analyses were similar in sensitivity analyses when using UKB as the outcome cohort for heart failure, atrial fibrillation, and coronary artery disease(compare Figure 6 and Figure S13 with Table S4). Furthermore, in a conservative approach using selectively genetic variants in the *ADIPOQ* locus in two-sample Mendelian randomization analyses results were similar(compare Figure 6 and Figure S13 with Figure S14).

In addition, we found no evidence of a non-linear effect of plasma adiponectin on the risk of heart failure, atrial fibrillation, aortic valve stenosis, or myocardial infarction using non-linear one-sample Mendelian randomization in the CGPS(Figures S15–18).

## 4 Discussion

We used observational and Mendelian randomization analyses to test our hypothesis that plasma adiponectin is associated observationally and causally, genetically with risk of heart failure, atrial fibrillation, aortic valve stenosis, and myocardial infarction. While elevated plasma adiponectin was associated with increased risk of heart failure, atrial fibrillation, aortic valve stenosis, and myocardial infarction in observational analyses, genetic one- and two-sample Mendelian randomization analyses could not support causality for these associations. Taken together, these findings are novel. Specific novel findings include the investigation of the causal role of plasma adiponectin in aortic valve stenosis and the use of individual participant data in Mendelian randomization facilitating exploration of subgroup associations and non-linear effects in the risk of heart failure, atrial fibrillation, aortic valve stenosis, and myocardial infarction.

Biologically, adiponectin potentially plays a pivotal role in cardiovascular disease by improving insulin sensitivity and exerting anti-atherogenic and anti-inflammatory properties, that is, theoretically, elevated adiponectin could protect against cardiovascular disease. However, higher plasma adiponectin has been associated with higher mortality in individuals with heart failure and associated cardiovascular diseases^41, 42^. This phenomenon has become known as the “adiponectin paradox”^41, 42^. In line with this, we found that elevated plasma adiponectin was associated with increased risk of heart failure and associated cardiovascular diseases in our observational analyses. Potential explanations for this counter intuitive relationship have been suggested to be a compensatory mechanism or a state of adiponectin resistance^41, 42^. However, our Mendelian randomization studies could not support a causal relationship, suggesting that adiponectin may be predominantly a biomarker or a bystander rather than a causal risk factor, although further research is needed to determine whether this holds throughout the adiponectin concentration range, especially in the lower concentration range. Thus, it remains possible that there could still be a protective causal association for variants leading to low adiponectin levels, and that these are harder to identify because of a counterregulatory increase in levels in the setting of comorbidities at higher adiponectin concentrations. In a two-sample Mendelian randomization analysis including 29,347 individuals, high N-terminal-pro-brain natriuretic peptide was associated with higher plasma adiponectin and the authors concluded that reverse causation potentially explained the adiponectin paradox in heart failure^43^. Interestingly, observational sensitivity analyses in our study did not indicate presence of reverse causation. Regarding residual confounding, we recently did a bidirectional one- and two-sample Mendelian randomization in 460,397 individuals with no indication of a causal interrelation between plasma adiponectin and BMI^5^. However, in another bidirectional two-sample Mendelian randomization with 210,088 individuals, abdominal fat accumulation was causally associated with low plasma adiponectin, while gluteofemoral fat was causally associated with high plasma adiponectin^4^. Furthermore, a genetic study found evidence of a causal relationship between insulin resistance and decreased plasma adiponectin^44^. Body fat distribution and insulin resistance could therefore be potential confounders.

Possible explanations as to why the present genetic findings differ from prior ample experimental work supporting insulin-sensitizing, anti-atherogenic, and anti-inflammatory roles of adiponectin^45, 46^ include the complexity of living humans compared with experimental settings by using in-vitro cells and animal models^44, 47^. Indeed, compensatory mechanisms known as canalization has previously been suggested as a potential explanation for observing such differences^48^. Alternatively, it has been argued that the relative small plasma adiponectin concentration differences observed in large genetic epidemiological studies may be without any clinical significance^49^. In this regard and with the caveat of being animal models, an early experimental study found that a two-fold increase in plasma adiponectin was required to decrease plasma glucose in mice administered with 28 μg/g body weight purified recombinant adiponectin^50^, while 1 μg/g recombinant adiponectin administered to wild-type and adiponectin-knockout mice reduced myocardial infarct size and apoptosis^51^.

Previous research has mainly investigated the observational association between plasma adiponectin and heart failure and have mostly suggested a positive association in a total of 5,574 individuals with 780 events^7, 8, 10–12^. Also, an observational meta-analysis with in total 18,558 individuals and 3,165 events found evidence of a positive association between plasma adiponectin and atrial fibrillation^9^. In contrast, no association was found between plasma adiponectin and coronary heart disease in another observational meta-analysis with in total 23,717 individuals^52^. Observationally, we found a positive association between elevated plasma adiponectin and heart failure, atrial fibrillation, aortic valve stenosis, and myocardial infarction by including up to 29,665 individuals with 3,746 events.

We conducted the first one-sample Mendelian randomization study on the association between plasma adiponectin with risk of heart failure, atrial fibrillation, aortic valve stenosis, and myocardial infarction facilitating exploration of non-linear effects and subgroup associations with no evidence of a causal effect. A recent two-sample Mendelian randomization in 547,261– 977,323 individuals concluded that elevated plasma adiponectin may be causally associated with reduced risk of coronary artery disease but not with heart failure and atrial fibrillation^53^. However, only one of four statistical methods could support the potential causal finding. Furthermore, they did not present calculations estimating power or instrument strength – a weak instrument can introduce bias towards the observational confounded estimate^54^. Differences compared with our one- and two-sample Mendelian randomization studies include the use of different exposure and outcome cohorts and choice of genetic variants. In our one-sample Mendelian randomization, we focused on genetic variants in and around the *ADIPOQ* and *CDH13* loci encoding respectively for plasma adiponectin and T-cadherin, a receptor recognizing adiponectin, while the former study included loci from the whole genome with the risk of horizontal pleiotropy. In support of the present one- and two-sample Mendelian randomization with up to 367,542 individuals on myocardial infarction, two-sample Mendelian randomization studies utilizing ADIPOGen, CARDIoGRAMplusC4D, and CARDIoGRAM with up to 281,422 individuals discovered no conclusive proof of a causal relationship between elevated plasma adiponectin and coronary artery disease and myocardial infarction^48, 55^. With our present study, we add by including aortic valve stenosis as an outcome, additional outcome cohorts, and one-sample Mendelian randomization studies with investigations of subgroup associations and non-linear effects.

Potential limitations in Mendelian randomization should be addressed. First, bias due to weak instruments. In one-sample Mendelian randomization, we used five genetic variants identified in genome-wide association studies with the lowest P-values and largest effect sizes in the association with plasma adiponectin^16–18^. Since our instrument only explained 3% of the variation in plasma adiponectin, we cannot rule out weak-instrument bias. However, with a F-value of 797 we have most likely limited the bias, arguing against a weak instrument bias as a major limitation in this study. Also, the bias should be towards the confounded observational association^54^. Furthermore, we used the ADIPOGen consortia with genetic variants explaining ~14% of the variation in plasma adiponectin in combination with HERMES, UKB, HUNT, deCODE, MGI, DiscovEHR, and the AFGen consortia in two-sample Mendelian randomization analyses with similar results. Second, population stratification bias. For one-sample Mendelian randomization we used an ethnically homogenous population; thus, bias due to population stratification is less likely. Moreover, since there was no evidence of Hardy-Weinberg disequilibrium, genotyping and population sampling errors also seem unlikely. Third, pleiotropy of the included genetic variants. In one-sample Mendelian randomization we selected biologically relevant genetic variants in the *ADIPOQ* and *CDH13* loci encoding respectively for plasma adiponectin and T-cadherin, a receptor recognizing adiponectin. Furthermore, we used different methods accounting for potential pleiotropy in sensitivity analyses in both one- and two-sample Mendelian randomization. Fourth, linkage disequilibrium. In one-sample Mendelian randomization we found no indication of linkage disequilibrium between the selected genetic variants^5, 6, 19, 20^, and genetic variants in two-sample Mendelian randomization were also pruned for linkage disequilibrium^27, 32^. Fifth, statistical power. Relatively large sample sizes are required to obtain sufficient statistical power in Mendelian randomization studies. Generally, the confidence intervals are wider in the CGPS compared with the UKB due to lower study power. Indeed, based on the 95% CIs for our causal odds ratios in the CGPS, an association is still possible for aortic valve stenosis in either direction. The confidence intervals for aortic valve stenosis from one- and two-sample Mendelian randomization are overlapping, and the estimates are largely similar, although the CGPS estimate is nominally slightly higher. This could partly be explained by ascertainment bias in the UKB due to shorter follow-up compared with the follow-up in the CGPS. Also, MR-Egger regression may give biased estimates when applied in one-sample Mendelian randomization. However, the bias of the MR-Egger estimate should be towards the confounded observational association and the bias is reduced when I^2^_GX_ is high, as in the present study with a I^2^_GX_ of 94–95%^56^. Lastly, we did not examine heart failure with preserved ejection fraction (HFpEF) and heart failure with reduced ejection fraction (HFrEF) separately, which have differences in pathophysiology^57^. Indeed, obesity and dysmetabolism appear to be stronger risk factors for HFpEF than HFrEF, hence the lack of differentiating between HFpEF and HFrEF could be a potential limitation. Since we use ICD-8 and ICD-10 codes defined before the introduction of HFpEF in 2023, our outcome includes for practical purposes only HFrEF.

The present study has several strengths. Importantly, we used both one- and two-sample Mendelian randomization analyses with large sample sizes, attenuating the risk of false associations. Furthermore, the observational and genetic one-sample Mendelian randomization were conducted in a single homogenous cohort and information on plasma adiponectin and outcome diagnoses were assessed with identical methods in all individuals. Moreover, with the availability of individual participant data in our one-sample Mendelian randomization, we were able to study non-linear effects and subgroup associations with no evidence of a causal effect, supporting the overall conclusions. If there were non-linearity it could be possible to find an incorrect null result in the main analyses due to the incorrect linearity assumption^30^. Thus, including the non-linear models shows that our null result is robust to relaxing that assumption. Although, the power of the analysis means there may still be a small undetected effect. Furthermore, subgroup analyses support that it is not confounding by covariates causing a false null result. Lastly, we used both a conservative Mendelian randomization approach including variants in and around the *ADIPOQ* and *CDH13* loci and a more liberal approach including variants from various regions, with similar results.

In conclusion, observationally, elevated plasma adiponectin was associated with increased risk of heart failure, atrial fibrillation, aortic valve stenosis, and myocardial infarction. However, genetic studies could not support causality for those associations.

## Supplementary Material

Supplementary material

## Figures and Tables

**Table 1:** Baseline characteristics in observational and genetic analyses in individuals in the Copenhagen General Population Study.

	Observational analyses		Genetic analyses	
	Individuals in observational analyses (N=30,045)	Association with plasma adiponectin, P-value*	Individuals in genetic analyses (N=96,903)	Association with genetic adiponectin score, P-value*
Age, years	62 (51-72)	<1 x 10^-300^	58 (48-67)	0.97
Women	15,328 (51)	<1 x 10^-300^	53,298 (55)	0.44
Hypertension	20,184 (67)	5 x 10^-10^	58,531 (60)	0.12
Diabetes	1,942 (6)	1 x 10^-53^	4,112 (4)	0.95
Lipid-lowering drugs	4,273 (14)	1 x 10^-17^	11,480 (12)	0.11
Current smoking	7,066 (24)	2 x 10^-41^	17,063 (18)	0.13
Alcohol consumption, units/week	9 (4-16)	1 x 10^-26^	8 (4-15)	0.86
Poor socioeconomic status	1,797 (6)	4 x 10^-29^	3,105 (3)	0.44
Physical inactivity	2,446 (8)	2 x 10^-11^	6,053 (6)	0.29
Body mass index, kg/m^2^	26.0 (23.5-28.9)	<1 x 10^-300^	25.6 (23.2-28.4)	0.95
Waist circumference, cm	92 (83-101)	<1 x 10^-300^	90 (80-99)	0.75
Non-HDL cholesterol, mmol/L	4.0 (3.2-4.8)	6 x 10^-171^	3.9 (3.2-4.7)	0.01^NS^
Plasma high-sensitive C-reactive protein, mg/L	1.6 (1.0-3.0)	8 x 10^-13^	1.4 (1.0-2.3)	0.34

Data summarized as median (25th-75th percentiles), or N (%).

* Calculated using linear or logistic regression, as appropriate. N=number. Non-HDL=non-high-density lipoprotein.

NS Not significant, that is, did not meet Bonferroni corrected significance level for multiple testing of P=0.05/13=0.004 instead of conventional P=0.05.

## Data Availability

Data from the Copenhagen General Population Study can be made locally accessible under controlled conditions from the corresponding author upon reasonable request. For two-sample Mendelian randomization analyses data are available through the MR-Base platform using GWAS Catalog^27^ or from the UKB^26^. In the present study, we used data on plasma adiponectin (ADIPOGen id:ieu-a-1), heart failure (HERMES id:ebi-a-GCST009541), and atrial fibrillation (AFGen id:ebi-a-GCST006414) from the MR-Base platform^27^, and aortic valve stenosis and myocardial infarction from the UKB^26^.

## Translational Perspective

Adiponectin, an adipocyte-secreted protein-hormone, exerts insulin-sensitizing, anti-atherogenic, and anti-inflammatory properties in preclinical studies. In clinical observational and genetic studies, the picture is less clear. To unravel if adiponectin plays a causal role in heart failure and associated cardiovascular diseases, we used an observational and genetic Mendelian randomization design. We found that elevated plasma adiponectin was associated observationally with increased risk of heart failure, atrial fibrillation, aortic valve stenosis, and myocardial infarction. However, genetic evidence did not support causality for those associations suggesting that adiponectin may be predominantly a biomarker or a bystander rather than a causal risk factor, although further research is needed to determine whether this holds throughout the concentration range of adiponectin, especially in the lower concentration range.
