# Supplementary material for: Plasma Adiponectin Levels and Risk of Heart Failure, Atrial Fibrillation, Aortic Valve Stenosis, and Myocardial Infarction: Large scale observational and Mendelian Randomization evidence
Source: Cardiovasc Res. Author manuscript; Available in PMC 2024 Feb 28. (PMC10898934; doi:10.1093/cvr/cvad162)
Supplement: Supplementary material [file EMS190937-supplement-Supplementary_material.docx]

**SUPPLEMENT**

**Plasma Adiponectin Levels and Risk of Heart Failure, Atrial Fibrillation, Aortic Valve Stenosis, and Myocardial Infarction: Large scale observational and Mendelian Randomization evidence**

Maria Booth Nielsen, MD; Yunus Çolak, MD, PhD, DMSc; Marianne Benn, MD, PhD, DMSc; Amy Mason, MMath, PhD; Stephen Burgess, MMath, PhD; and Børge Grønne Nordestgaard, MD, DMSc

**Cohorts for two-sample Mendelian randomization**

***Information on adiponectin***

**ADIPOGen**

The ADIPOGen Consortium identifies genetic variation associated with plasma adiponectin with information on 29,347 European ancestry^1^.

***Information on heart failure***

**HERMES**

*Heart Failure Molecular Epidemiology for Therapeutic Targets* (HERMES) investigates the genetics of heart failure. We included information on 977,323 individuals of European ancestry with 47,309 heart failure cases^2^.

***Information on aortic valve stenosis and myocardial infarction***

**UK Biobank**

The UK Biobank recruited 500,000 individuals across the general population in the United Kingdom aged 40-69 at inclusion in 2006-2010^3^. The study was approved by the Northwest Haydock Research Ethics Committee(16/NW/0274). We included information on 367,561 British individuals with 3,528 aortic valve stenosis cases (ICD-10:I35 and I35.2) and 12,339 myocardial infarction cases (ICD-9: 410.X and ICD-10: I21.X and I22.X). Accessed on November 2021. Genetic associations with outcomes were estimated using logistic regression with adjustment for age, sex, and 10 genomic principal components to account for population structure. The analytic sample was defined using a previously described approach^4^: we excluded participants with genetic sex mismatch, non-European ancestries (self-report or inferred by genetics), or excess heterozygosity (>3 standard deviations from the mean) and included only one of each set of related participants (third-degree relatives or closer).

***Information on atrial fibrillation***

From a genome-wide association study (GWAS) with information on 1,030,836 individuals with 60,620 atrial fibrillation cases from the studies listed below – summary from the original GWAS^5^:

**HUNT**

*The Nord-Trøndelag Health Study* (HUNT) is a population-based health survey conducted in the county of Nord-Trøndelag, Norway. We included information on 69,635 Norwegians with 6,493 atrial fibrillation cases in a combination of hospital, out-patient, and emergency room discharge diagnoses (ICD-9 and ICD-10).

**deCODE**

deCODE is a company located in Iceland that investigates variations in the genome associated with common diseases. We included information on 371,632 Icelanders with 13,471 atrial fibrillation cases (ICD-10 code I48 and ICD-9 code 427.3).

**MGI**

The Michigan Genomics Initiative (MGI) combines patient electronic health record data with genetic data. We included information on 1,226 atrial fibrillation cases (ICD-9 billing code 427.31).

**DiscovEHR**

The DiscovEHR collaboration cohort is a hospital-based cohort. We included information on 48,482 individuals of European ancestry with 6,679 atrial fibrillation cases (ICD-10 I48).

**AFGen**

Atrial fibrillation association summary statistics. We included information on 133,073 individuals with 17,931 atrial fibrillation cases.

**UK Biobank** (ICD-9 427.3 and ICD-10 I48)

**Table S1.** **Listing of genetic variants (SNPs) used in one- and two-sample Mendelian randomization.**

| **SNPs in two-sample MR** | **Gene** | **Chr** | **EA** | **NEA** | **EAF in EUR** | **Reported associations with potential confounders** | **SNPs in one-sample MR** |
| --- | --- | --- | --- | --- | --- | --- | --- |
|  | *LINC02043* | 3 | T | C | 0.48 |  | rs266717**^‡^** |
|  | *CMIP^**^* | 16 | T | C | 0.29 | HDL-Cholesterol, BMI adjusted WHR | rs2925979 |
| rs1108842 | *GNL3* | 3 | A | C | 0.48 | Waist-hip-ratio, Body mass index |  |
| rs12051272 | *CDH13* | 16 | G | T | 0.98 |  |  |
| rs1597466 | *SIAH2* | 3 | G | T | 0.90 | Trunk fat |  |
| **rs17366568***^‡^ | *ADIPOQ* | 3 | G | A | 0.89 |  | **rs17366568***^‡^ |
| **rs2062632***^‡^ | *KNG1* | 3 | T | C | 0.75 |  | **rs2062632***^‡^ |
| rs2927324 | *CMIP* | 16 | T | C | 0.55 |  |  |
| rs601339 | *RP11-324E6.6* | 12 | G | A | 0.18 |  |  |
| **rs6810075***^‡^ | *ADIPOQ* | 3 | T | C | 0.60 |  | **rs6810075***^‡^ |
| rs731839 | *PEPD* | 19 | G | A | 0.33 | Fat percentage |  |
| rs7615090**^‡^** | *TMEM207* | 3 | G | T | 0.14 |  |  |
| rs7955516 | *RP11-284H19.1* | 12 | C | A | 0.38 |  |  |
| rs8042532 | *LOXL1* | 15 | G | T | 0.03 |  |  |

Information on single nucleotide polymorphisms (SNPs) retrieved from <http://www.phenoscanner.medschl.cam.ac.uk> ^6, 7^ and <https://www.ensembl.org>. *^*^*Indicate the overlapping SNPs used in both one- and two-sample Mendelian randomization. Unfortunately, we were not able to find rs266717 and rs2925979 from the one-sample Mendelian randomization in the two-sample Mendelian randomization outcome cohorts. ^**^In the *CDH13* locus. ^‡^SNPs in the *ADIPOQ* gene or locus. Chr=chromosome. EA=effect allele. EAF=effect allele frequency. EUR=European population. NEA=non effect allele/other allele. MR=Mendelian randomization. SNPs=single nucleotide polymorphisms.

| **SNP** | | **D**' | | | |
| --- | --- | --- | --- | --- | --- |
|  |  | rs2062632 | rs266717 | rs6810075 | rs17366568 |
| **R^2^** | rs2062632 |  | 0.108 | 0.063 | 0.015 |
|  | rs266717 | 0.004 |  | 0.615 | 0.212 |
|  | rs6810075 | 0.001 | 0.236 |  | 0.099 |
|  | rs17366568 | 0.000 | 0.005 | 0.002 |  |

**Table S2.** **Pairwise Linkage Disequilibrium Coefficients D' and R^2^ in a European population of genetic variants (SNPs) in the *ADIPOQ* locus used in one-sample Mendelian randomization analyses.**

| **R^2^ and D**' | 0 | 0.1 | 0.2 | 0.3 | 0.4 | 0.5 | 0.6 | 0.7 | 0.8 | 0.9 | 1 |
| --- | --- | --- | --- | --- | --- | --- | --- | --- | --- | --- | --- |
| **Correlation** | Low-------------------------------------------------------------------------------------------------High | | | | | | | | | | |

Information on linkage disequilibrium from a web-based LD-link application ^8^. D prime (D') is an indicator of allelic segregation for two genetic variants. D' values range from 0 to 1 with higher values indicating tight linkage of alleles. A D' value of 0 indicates no linkage of alleles. D' is shown above diagonal of empty cells. R squared (R^2^) is a measure of correlation of alleles for two genetic variants. R^2^ values range from 0 to 1 with higher values indicating a higher degree of correlation. R^2^ is shown below diagonal of empty cells. SNPs=Single nucleotide polymorphisms.
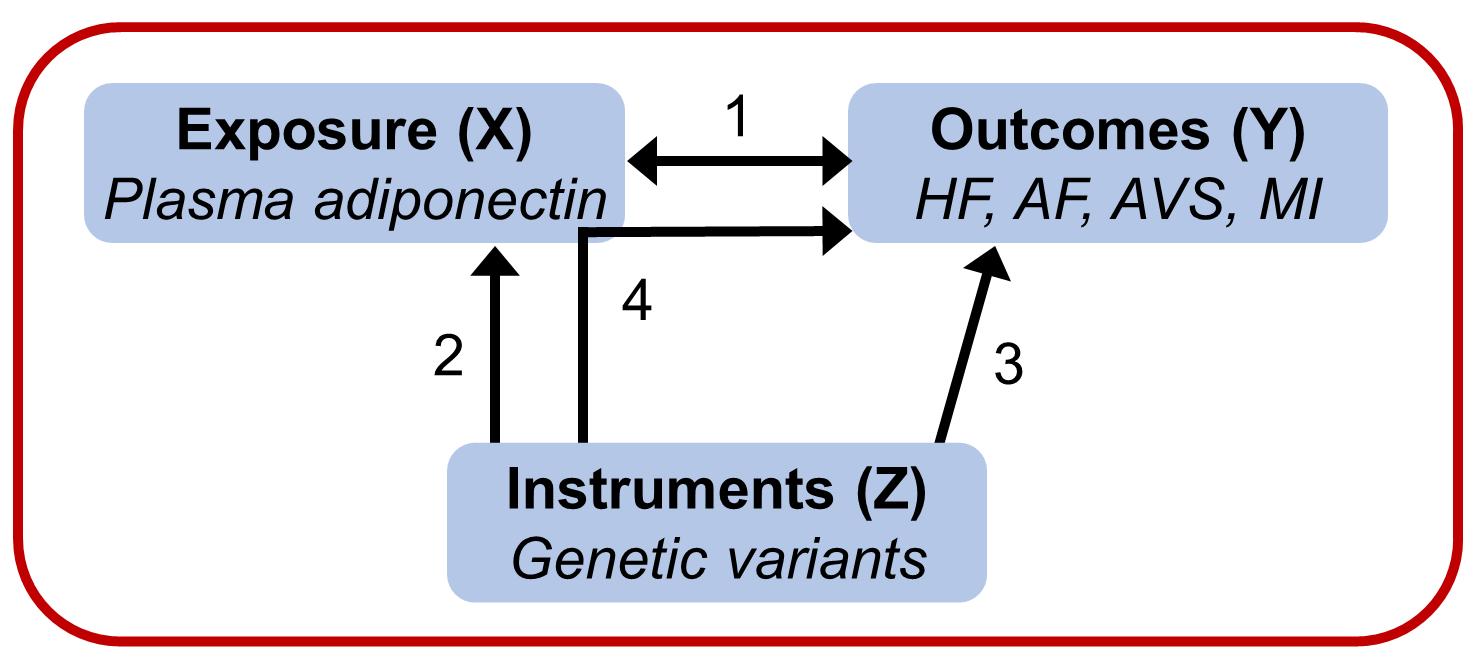


**Figure S1. Testing the observational and causal relationship of plasma adiponectin with heart failure (HF), atrial fibrillation (AF), aortic valve stenosis (AVS), and myocardial infarction (MI) in the Copenhagen General Population Study.** Step 1: The associations between plasma adiponectin as the exposure (X) and HF, AF, AVS, and MI as the outcomes (Y) were investigated. Step 2: The association between genetic variants as the instruments (Z) and plasma adiponectin as the exposure (X) was investigated. Step 3: The associations between genetic variants as the instruments (Z) and HF, AF, AVS, and MI as the outcomes (Y) were investigated. Step 4: The associations between genetically determined plasma adiponectin as the exposure-instrument (X+Z) and HF, AF, AVS, and MI as the outcomes (Y) were investigated (Mendelian randomization analysis to estimate the causal effect of plasma adiponectin on HF, AF, AVS, and MI). Observational analyses in Step 1 were multivariable adjusted for age, sex, hypertension, diabetes, use of lipid-lowering drugs, smoking status, socioeconomic status, physical activity, BMI, waist circumference, and plasma non-HDL cholesterol and high-sensitive C-reactive protein. Genetic analyses (Steps 2–4) were adjusted for age and sex.

**Table S3. Power calculation for Mendelian randomization analyses.**

| **Exposure cohort** | **Outcome cohort** | **Sample size/events** | **Type-I**  **error rate** | **SNPs** | **Variance in exposure explained by SNPs (R^2^)** | **Causal odds ratio with 80% power to detect per 1 SD^*^** | **Causal odds ratio with 80% power to detect per 1 unit^**^** |
| --- | --- | --- | --- | --- | --- | --- | --- |
| ***Two-sample*** | | | | | | | |
| ADIPOGen | HERMES  (heart failure) | 977,323/47,309 | 0.05 | 12 | 0.14 | 1.036 | 1.073 |
| ADIPOGen | HUNT, deCODE, MGI, DiscovEHR, UKB, AFGen (atrial fibrillation) | 1,030,836/60,620 | 0.05 | 12 | 0.14 | 1.032 | 1.065 |
| ADIPOGen | UKB  (aortic valve stenosis) | 367,561/3,528 | 0.05 | 12 | 0.14 | 1.136 | 1.289 |
| ADIPOGen | UKB  (myocardial infarction) | 361,194/7,018 | 0.05 | 12 | 0.14 | 1.095 | 1.198 |
| ***One-sample*** | | | | | | | |
| CGPS | CGPS  (heart failure) | 96,903/4,599 | 0.05 | 5 | 0.02 | 1.349 | 1.815 |
| CGPS | CGPS  (atrial fibrillation) | 96,903/9,020 | 0.05 | 5 | 0.02 | 1.245 | 1.547 |
| CGPS | CGPS  (aortic valve stenosis) | 96,903/1,528 | 0.05 | 5 | 0.02 | 1.667 | 2.766 |
| CGPS | CGPS  (myocardial infarction) | 96,903/4,449 | 0.05 | 5 | 0.02 | 1.356 | 1.834 |

Power calculations were performed using Burgess’ online power calculator (<https://sb452.shinyapps.io/power/>)^9^. The standard deviation was calculated based on data from CGPS (SD=0.5023101).*Causal effect (odds ratio, exp(β_1_)) per SD change in log-transformed plasma adiponectin.**Causal effect (odds ratio, exp(β_1_)) per 1 unit change in log-transformed plasma adiponectin. CGPS=Copenhagen General Population Study. HERMES=Heart Failure Molecular Epidemiology for Therapeutic Targets. HUNT=The Nord-Trøndelag Health Study. MGI=The Michigan Genomics Initiative. UKB=UK Biobank. SNPs=Single nucleotide polymorphisms. SD=standard deviation.


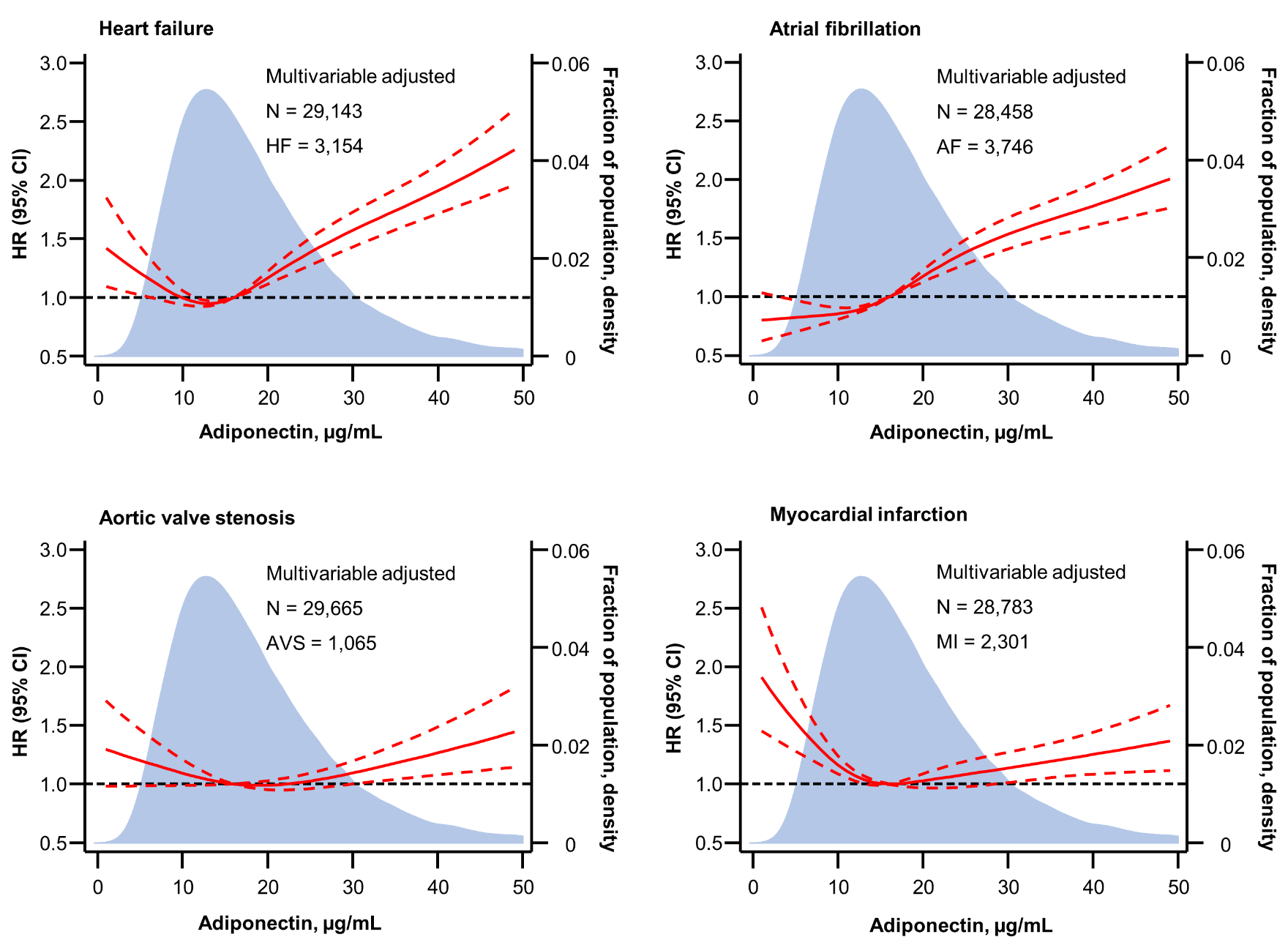


**Figure S2. Observational association of plasma adiponectin with heart failure (HF), atrial fibrillation (AF), aortic valve stenosis (AVS), and myocardial infarction (MI) in the Copenhagen General Population Study using a less adjusted model.** Hazard ratios (HR) are indicated with a solid red line and 95% confidence interval (CI) with dashed red lines. Median concentration of plasma adiponectin (16 µg/mL) was used as reference with a hazard ratio of 1.0 indicated with horizontal dashed black line. Individuals in the upper 1st percentile for plasma adiponectin (plasma adiponectin ≥50 µg/mL) were included in the analyses but excluded from the graphs for visual purposes. Analyses were multivariable adjusted for age (as timescale), sex, hypertension, smoking status, socioeconomic status, physical activity, body mass index, and waist circumference. Fraction of the population indicated with light blue. N=number of individuals.


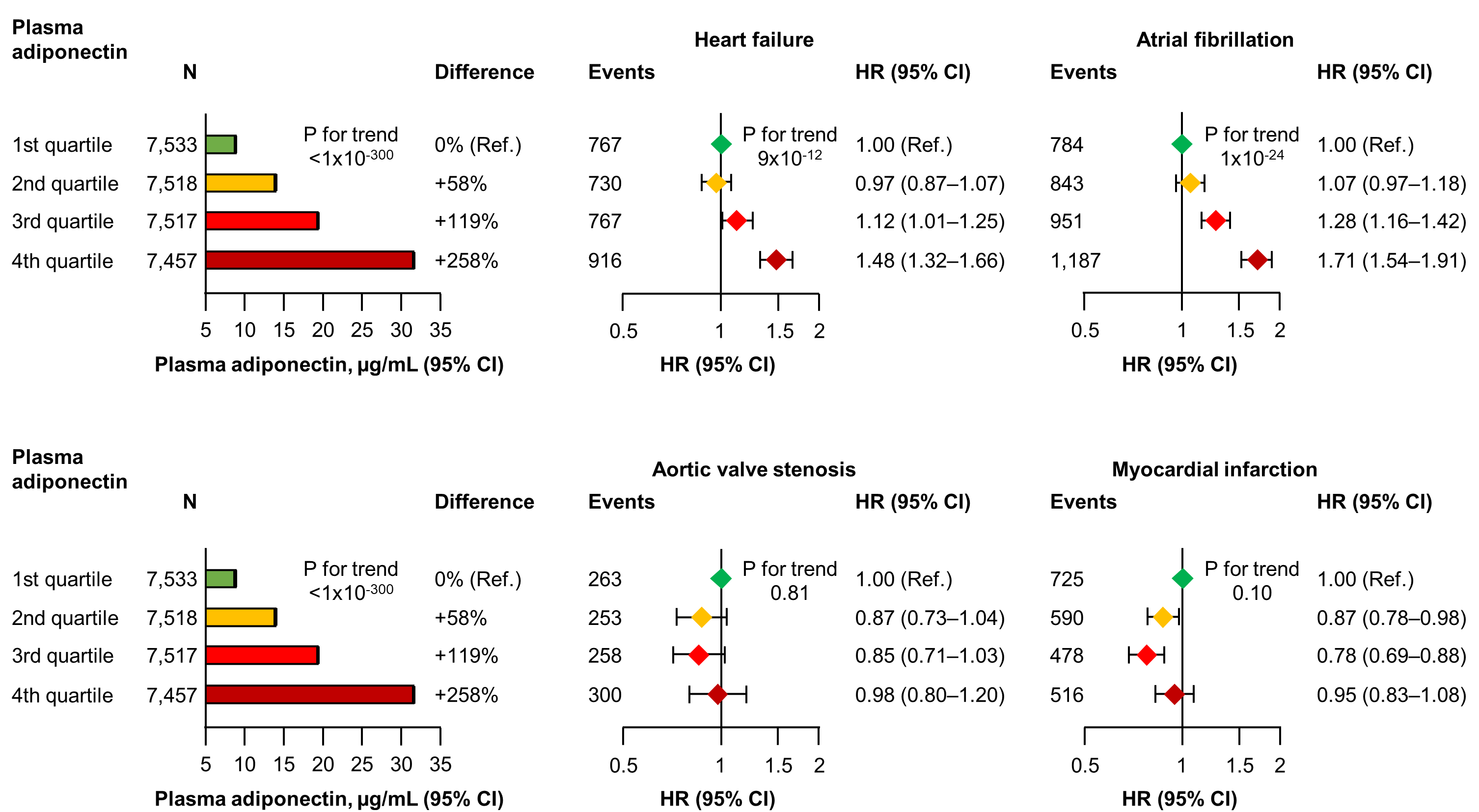


**Figure S3. Observational association of plasma adiponectin with heart failure, atrial fibrillation, aortic valve stenosis, and myocardial infarction in the Copenhagen General Population Study using a less adjusted model.** Geometric mean with 95% confidence interval (CI) for plasma adiponectin is indicated with bars and whiskers. Hazard ratios (HR) with 95% CI for heart failure, atrial fibrillation, aortic valve stenosis, and myocardial infarction are indicated with diamonds and whiskers. Analyses were multivariable adjusted for age (as timescale), sex, hypertension, smoking status, socioeconomic status, physical activity, body mass index, and waist circumference. N=number of individuals.


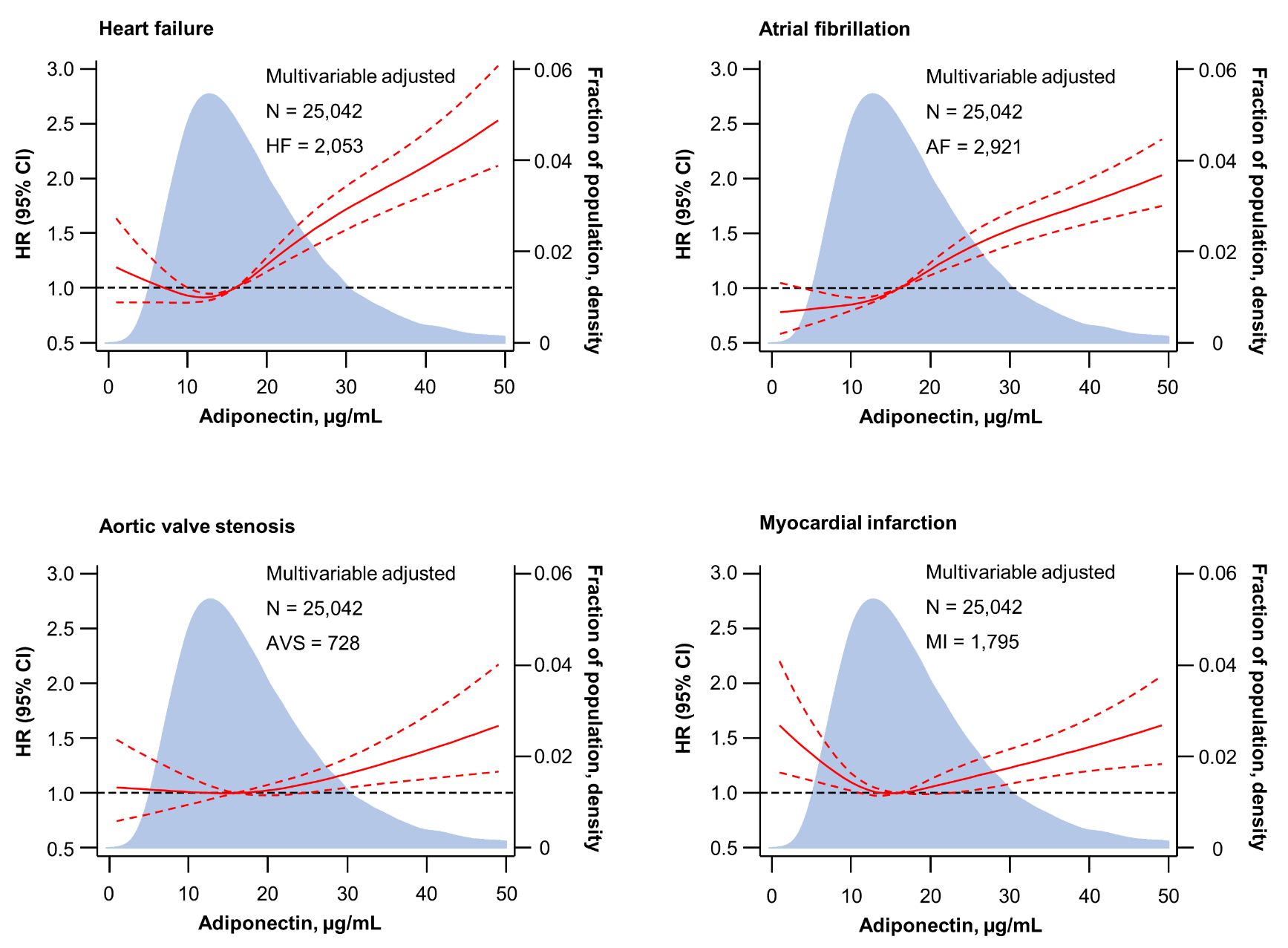


**Figure S4. Observational association of plasma adiponectin with heart failure (HF), atrial fibrillation (AF), aortic valve stenosis (AVS), and myocardial infarction (MI) in the Copenhagen General Population Study in individuals without prior cardiovascular disease.** Individuals with any prevalent cardiovascular disease, that is, HF, AF, AVS, MI, ischemic heart disease, ischemic stroke, or stroke, were excluded prior to analyses. Hazard ratios (HR) are indicated with solid red line and 95% confidence interval (CI) with dashed red lines. Median concentration of plasma adiponectin (16 µg/mL) was used as reference with a hazard ratio of 1.0 indicated with horizontal dashed black line. Individuals in the upper 1st percentile for plasma adiponectin (plasma adiponectin ≥50 µg/mL) were included in the analyses but excluded from the graphs for visual purposes. Analyses were multivariable adjusted for age, sex, hypertension, diabetes, use of lipid-lowering drugs, smoking status, socioeconomic status, physical activity, body mass index, waist circumference, non-high-density lipoprotein cholesterol, and plasma high-sensitive C-reactive protein. Fraction of the population indicated with light blue. N=number of individuals.


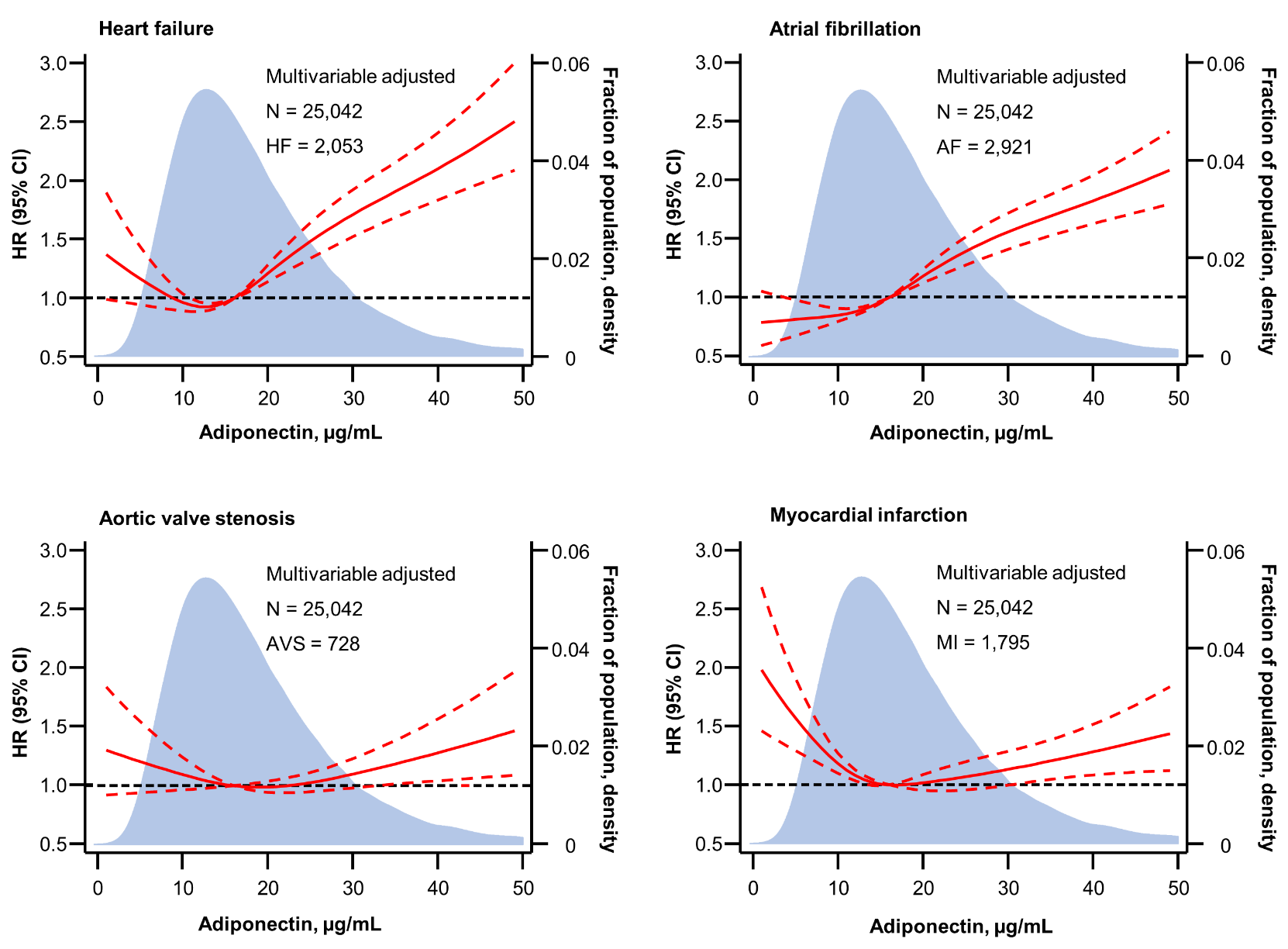


**Figure S5. Observational association of plasma adiponectin with heart failure (HF), atrial fibrillation (AF), aortic valve stenosis (AVS), and myocardial infarction (MI) in the Copenhagen General Population Study without prior cardiovascular disease in a less adjusted model.** Individuals with any prevalent cardiovascular disease, that is, HF, AF, AVS, MI, ischemic heart disease, ischemic stroke, or stroke, were excluded prior to analyses. Hazard ratios (HR) are indicated with solid red line and 95% confidence interval (CI) with dashed red lines. Median concentration of plasma adiponectin (16 µg/mL) was used as reference with a hazard ratio of 1.0 indicated with horizontal dashed black line. Individuals in the upper 1st percentile for plasma adiponectin (plasma adiponectin ≥50 µg/mL) were included in the analyses but excluded from the graphs for visual purposes. Analyses were multivariable adjusted for age, sex, hypertension, smoking status, socioeconomic status, physical activity, body mass index, and waist circumference. Fraction of the population indicated with light blue. N=number of individuals.


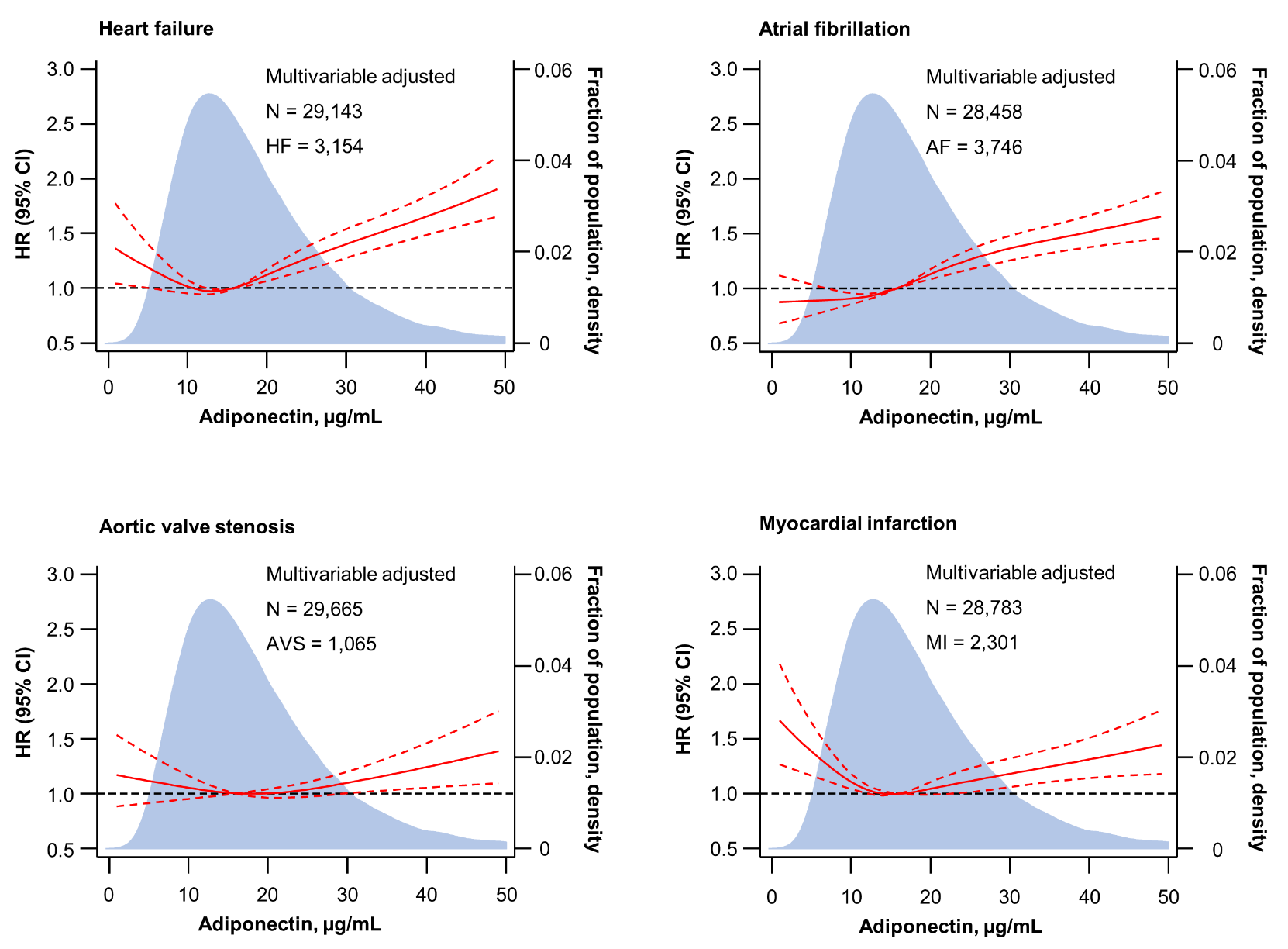


**Figure S6. Observational association of plasma adiponectin with heart failure (HF), atrial fibrillation (AF), aortic valve stenosis (AVS), and myocardial infarction (MI) in the Copenhagen General Population Study without adjustment for body mass index and waist circumference.** Hazard ratios (HR) are indicated with solid red line and 95% confidence interval (CI) with dashed red lines. Median concentration of plasma adiponectin (16 µg/mL) was used as reference with a hazard ratio of 1.0 indicated with horizontal dashed black line. Individuals in the upper 1st percentile for plasma adiponectin (plasma adiponectin ≥50 µg/mL) were included in the analyses but excluded from the graphs for visual purposes. Analyses were multivariable adjusted for age, sex, hypertension, diabetes, use of lipid-lowering drugs, smoking status, socioeconomic status, physical activity, non-high-density lipoprotein cholesterol, and plasma high-sensitive C-reactive protein. Fraction of the population indicated with light blue. N=number of individuals.


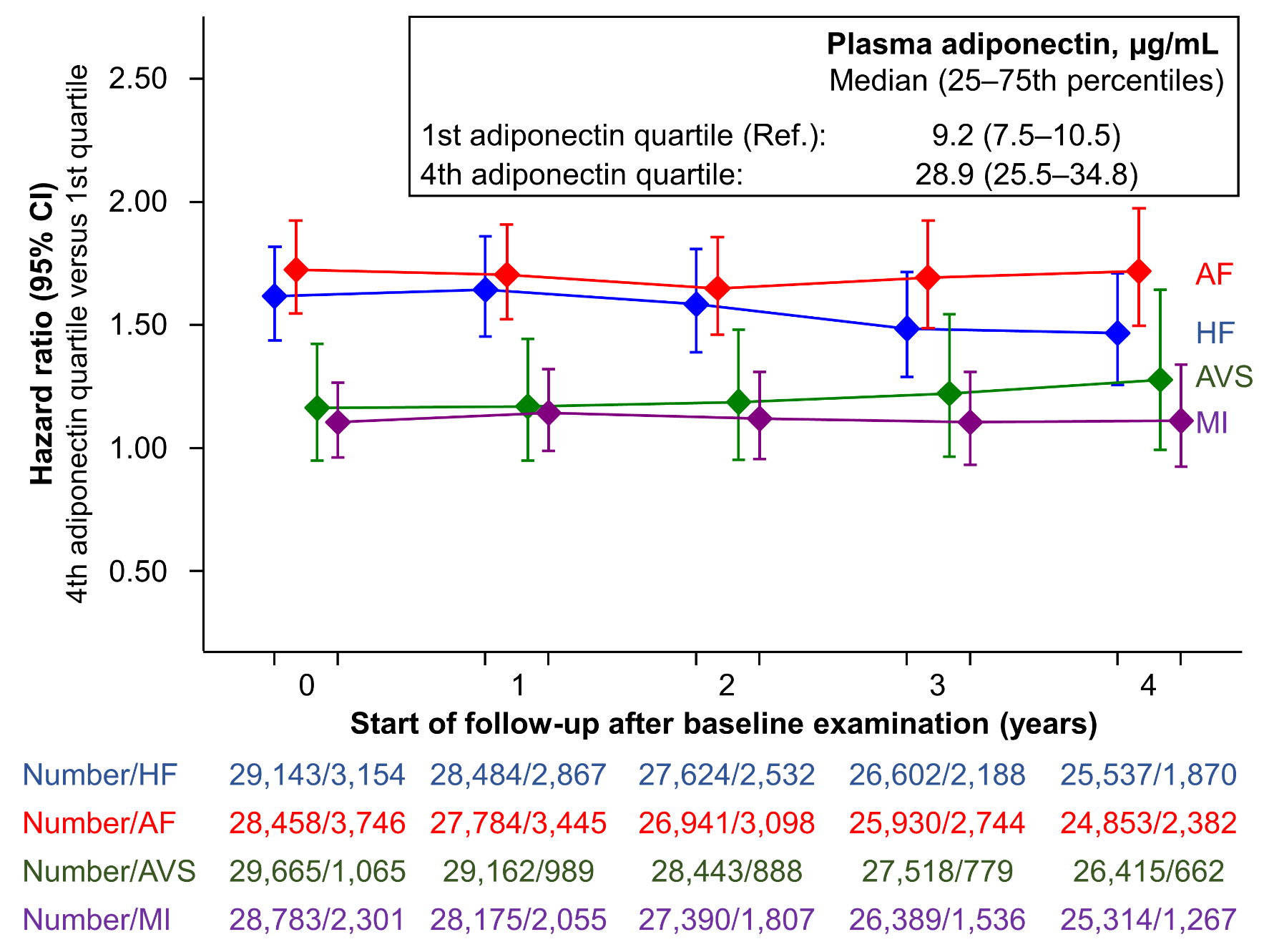


**Figure S7. Observational associations of plasma adiponectin with heart failure, atrial fibrillation, aortic valve stenosis, and myocardial infarction in the Copenhagen General Population Study.** Hazard ratios indicated with diamonds and 95% confidence interval (CI) when comparing individuals in the 4th *versus* 1st quartile of plasma adiponectin at 0–4 years after inclusion. Individuals with prior events were excluded from analyses. Hazard ratios for heart failure (HF) in blue, atrial fibrillation (AF) in red, aortic valve stenosis (AVS) in green, and myocardial infarction (MI) in purple.


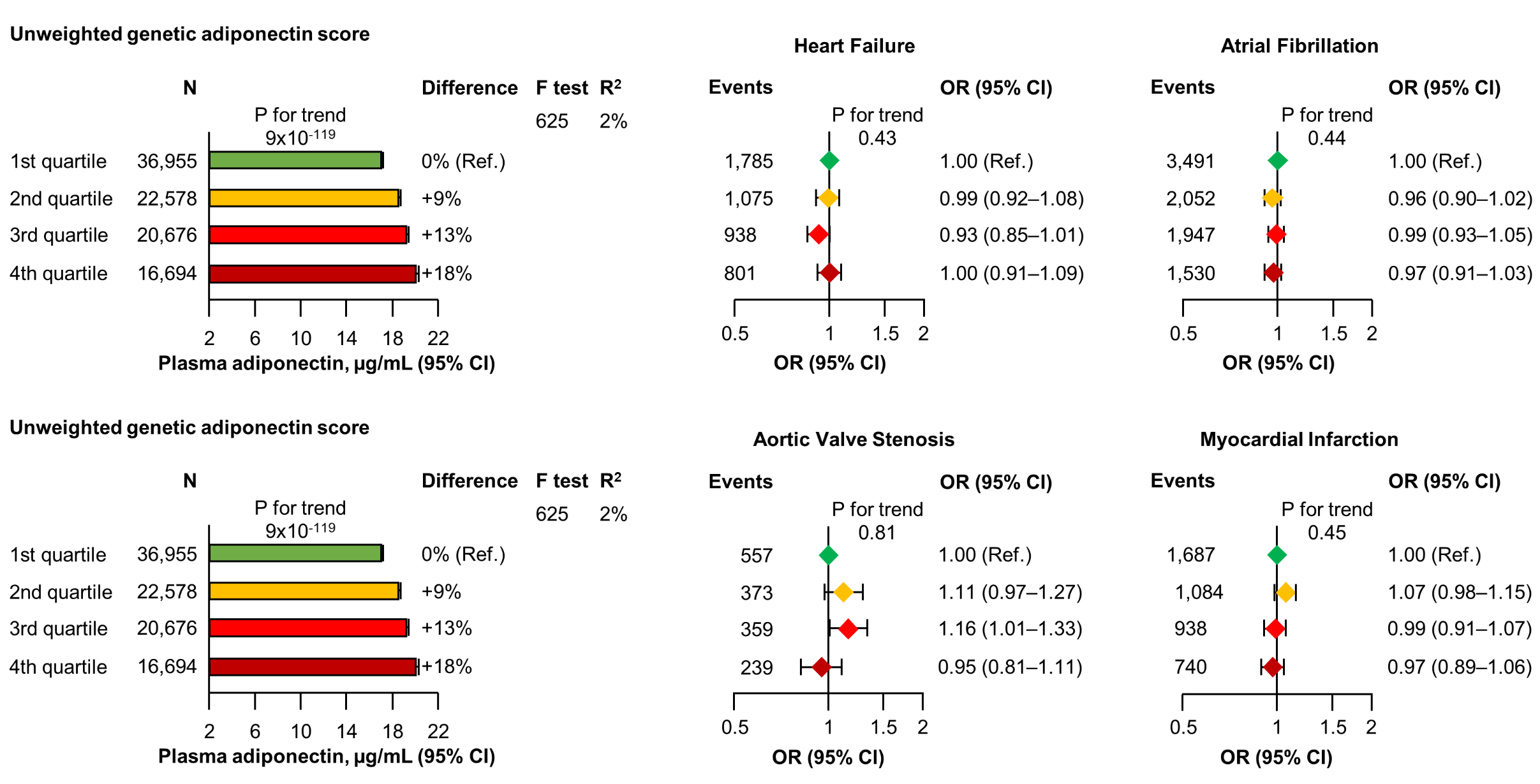


**Figure S8.** **Unweighted genetic association of plasma adiponectin with heart failure, atrial fibrillation, aortic valve stenosis, and myocardial infarction in the Copenhagen General Population Study.** Geometric mean with 95% confidence interval (CI) for plasma adiponectin is indicated with bars and whiskers. Odds ratio (OR) with 95% CI for heart failure atrial fibrillation, aortic valve stenosis, and myocardial infarction are indicated with diamonds and whiskers. Analyses were adjusted for age and sex. The unweighted genetic adiponectin score explained 2% of the variation in plasma adiponectin (R^2^) with an F-value of 625. N=number of individuals.


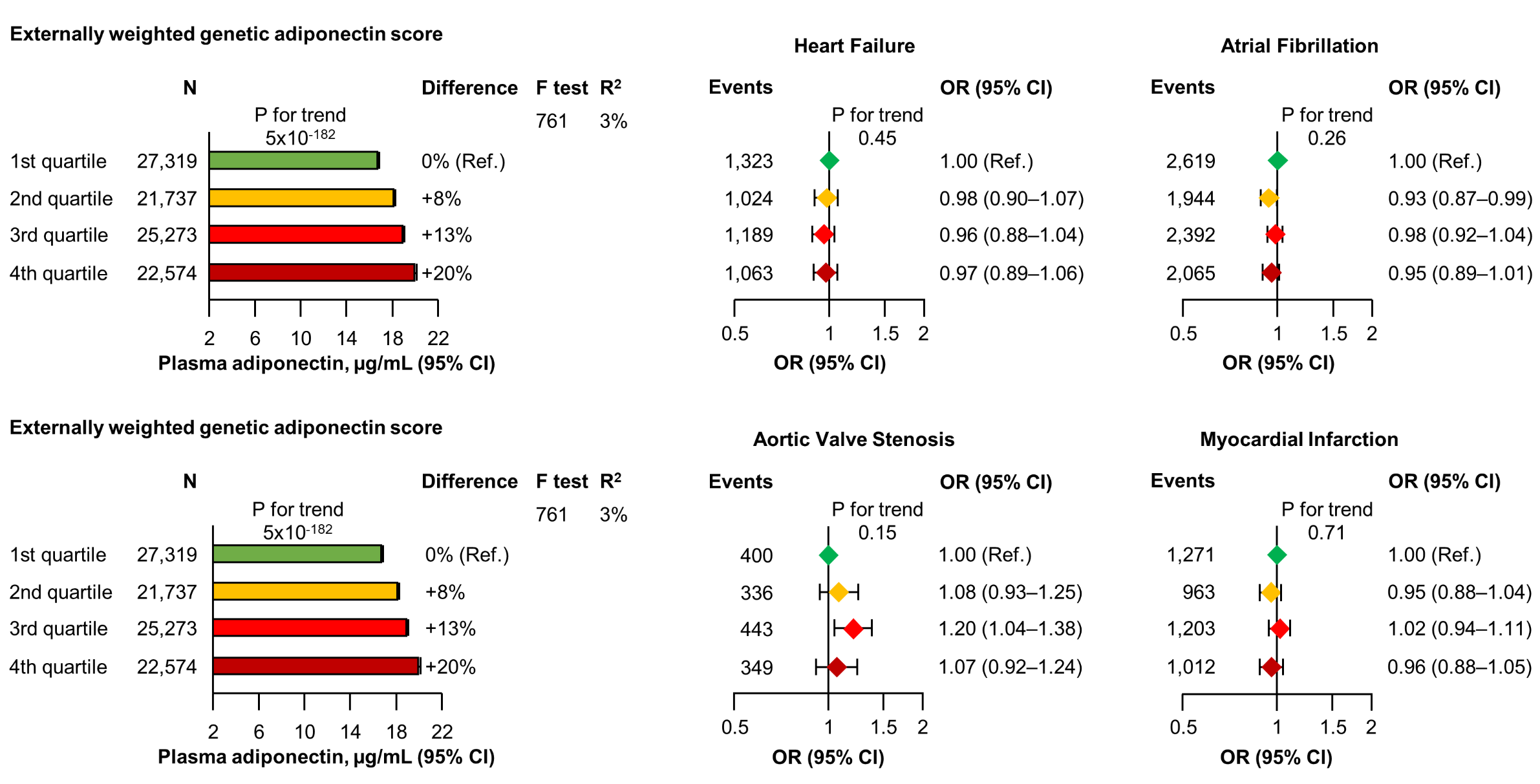


**Figure S9. Externally weighted genetic association of plasma adiponectin with heart failure, atrial fibrillation, aortic valve stenosis, and myocardial infarction in the Copenhagen General Population Study.** Geometric mean with 95% confidence interval (CI) for plasma adiponectin is indicated with bars and whiskers. Odds ratio (OR) with 95% CI for heart failure atrial fibrillation, aortic valve stenosis, and myocardial infarction are indicated with diamonds and whiskers. Analyses were adjusted for age and sex. The externally weighted genetic adiponectin score was created using coefficients from the ADIPOGen consortia^1^ explained 3% of the variation in plasma adiponectin (R^2^) with an F-value of 761. N=number of individuals.


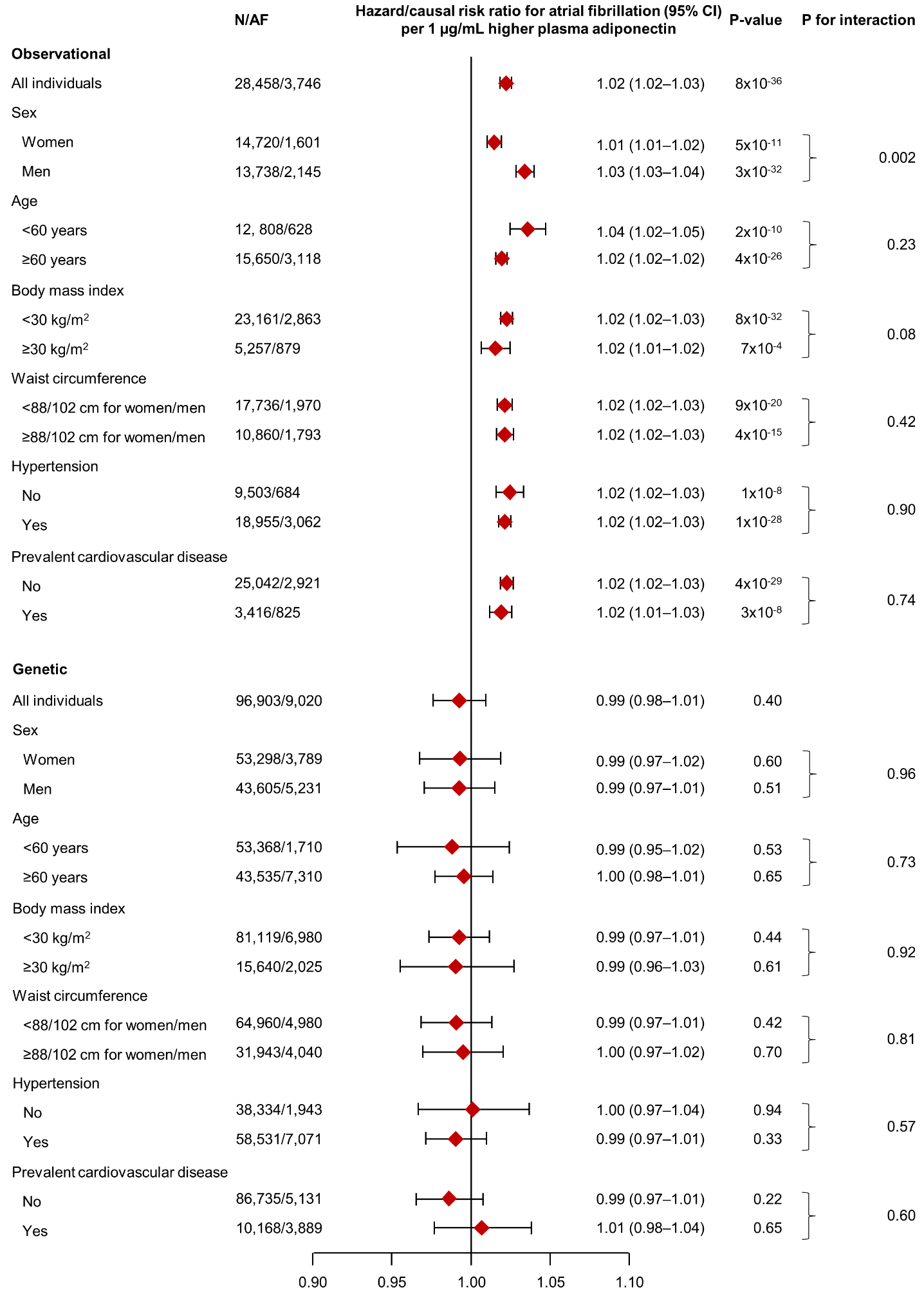


**Figure S10. Observational and genetic association of plasma adiponectin with atrial fibrillation (AF) in the Copenhagen General Population Study.** Observational analyses were multivariable adjusted for age, sex, hypertension, diabetes, use of lipid-lowering drugs, smoking status, socioeconomic status, physical activity, body mass index, waist circumference, non-high-density lipoprotein cholesterol, and plasma high-sensitive C-reactive protein. Genetic analyses used internal weighted genetic adiponectin score generated using genetic variations in the *ADIPOQ* (*KNG1* rs2062632, *LINC02043* rs266717, *ADIPOQ* rs6810075, and *ADIPOQ* rs17366568) and *CDH13* (*CMIP* rs2925979) loci and were adjusted for age and sex. AF=atrial fibrillation. N=number of individuals.


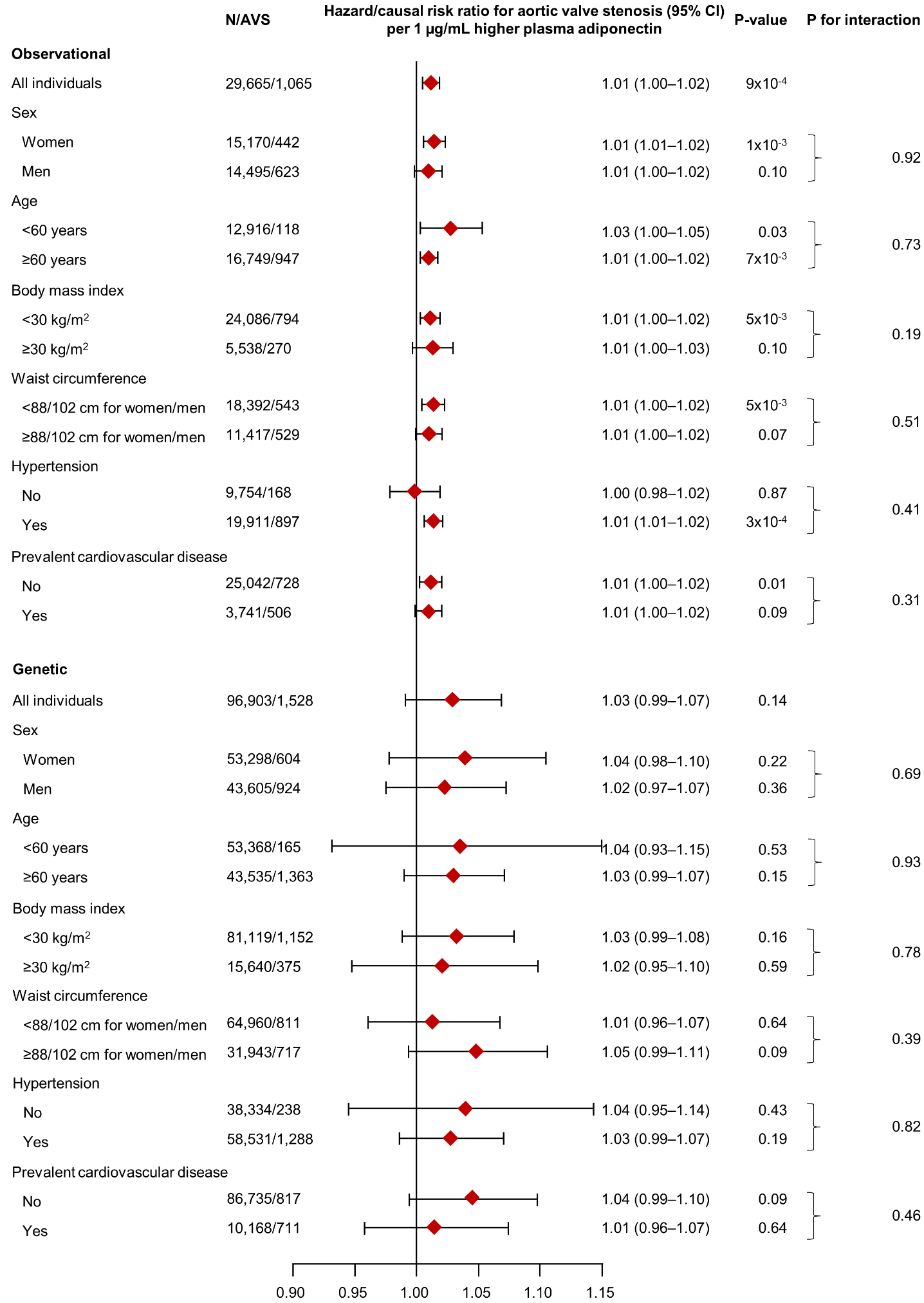


**Figure S11. Observational and genetic association of plasma adiponectin with aortic valve stenosis (AVS) in the Copenhagen General Population Study.** Observational analyses were multivariable adjusted for age, sex, hypertension, diabetes, use of lipid-lowering drugs, smoking status, socioeconomic status, physical activity, body mass index, waist circumference, non-high-density lipoprotein cholesterol, and plasma high-sensitive C-reactive protein. Genetic analyses used internal weighted genetic adiponectin score generated using genetic variations in the *ADIPOQ* (*KNG1* rs2062632, *LINC02043* rs266717, *ADIPOQ* rs6810075, and *ADIPOQ* rs17366568) and *CDH13* (*CMIP* rs2925979) loci and were adjusted for age and sex. AVS=aortic valve stenosis. N=number of individuals.


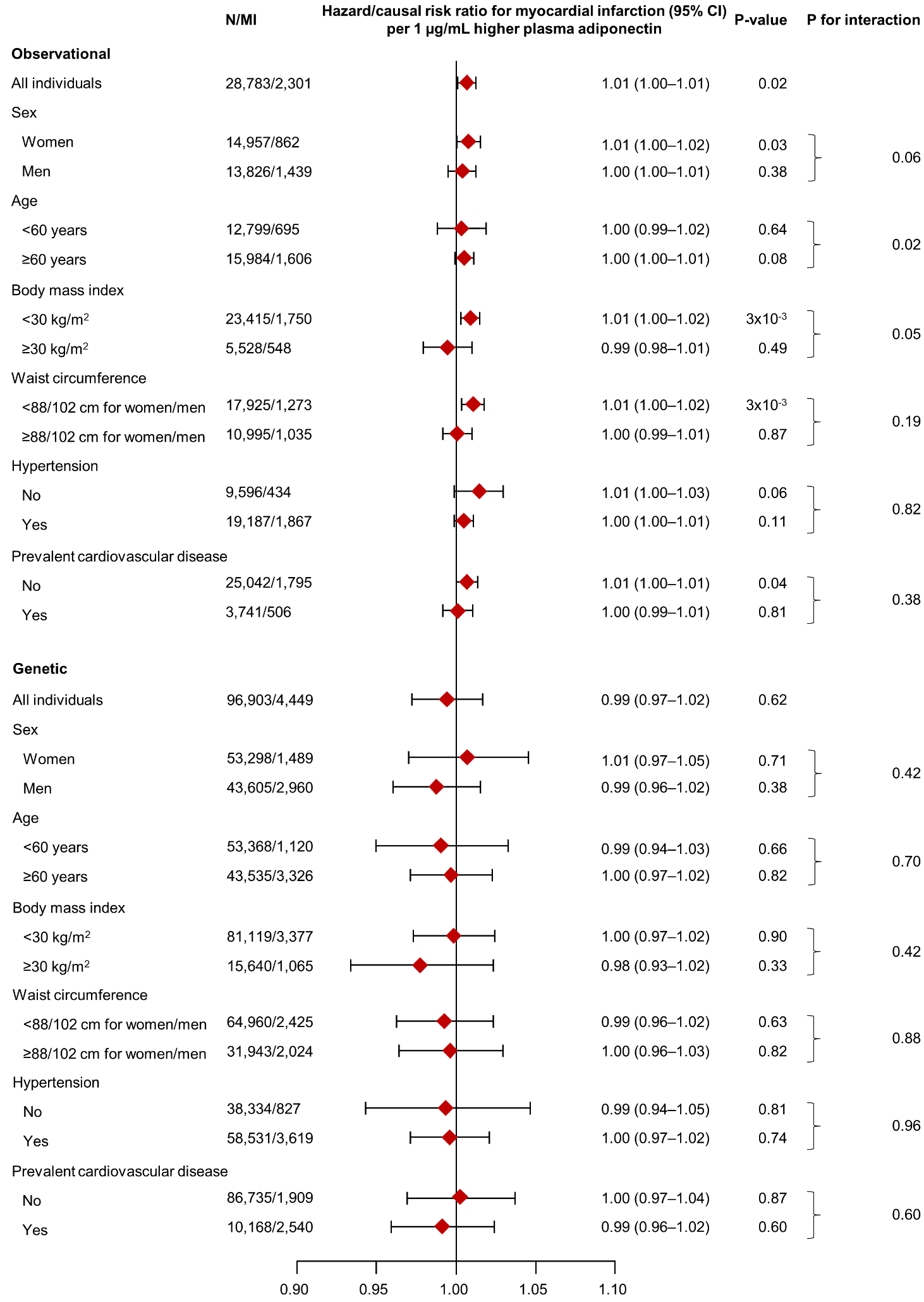


**Figure S12. Observational and genetic association of plasma adiponectin with myocardial infarction (MI) in the Copenhagen General Population Study.** Observational analyses were multivariable adjusted for age, sex, hypertension, diabetes, use of lipid-lowering drugs, smoking status, socioeconomic status, physical activity, body mass index, waist circumference, non-high-density lipoprotein cholesterol, and plasma high-sensitive C-reactive protein. Genetic analyses used internal weighted genetic adiponectin score generated using genetic variations in the *ADIPOQ* (*KNG1* rs2062632, *LINC02043* rs266717, *ADIPOQ* rs6810075, and *ADIPOQ* rs17366568) and *CDH13* (*CMIP* rs2925979) loci and were adjusted for age and sex. MI=myocardial infarction. N=number of individuals.

**
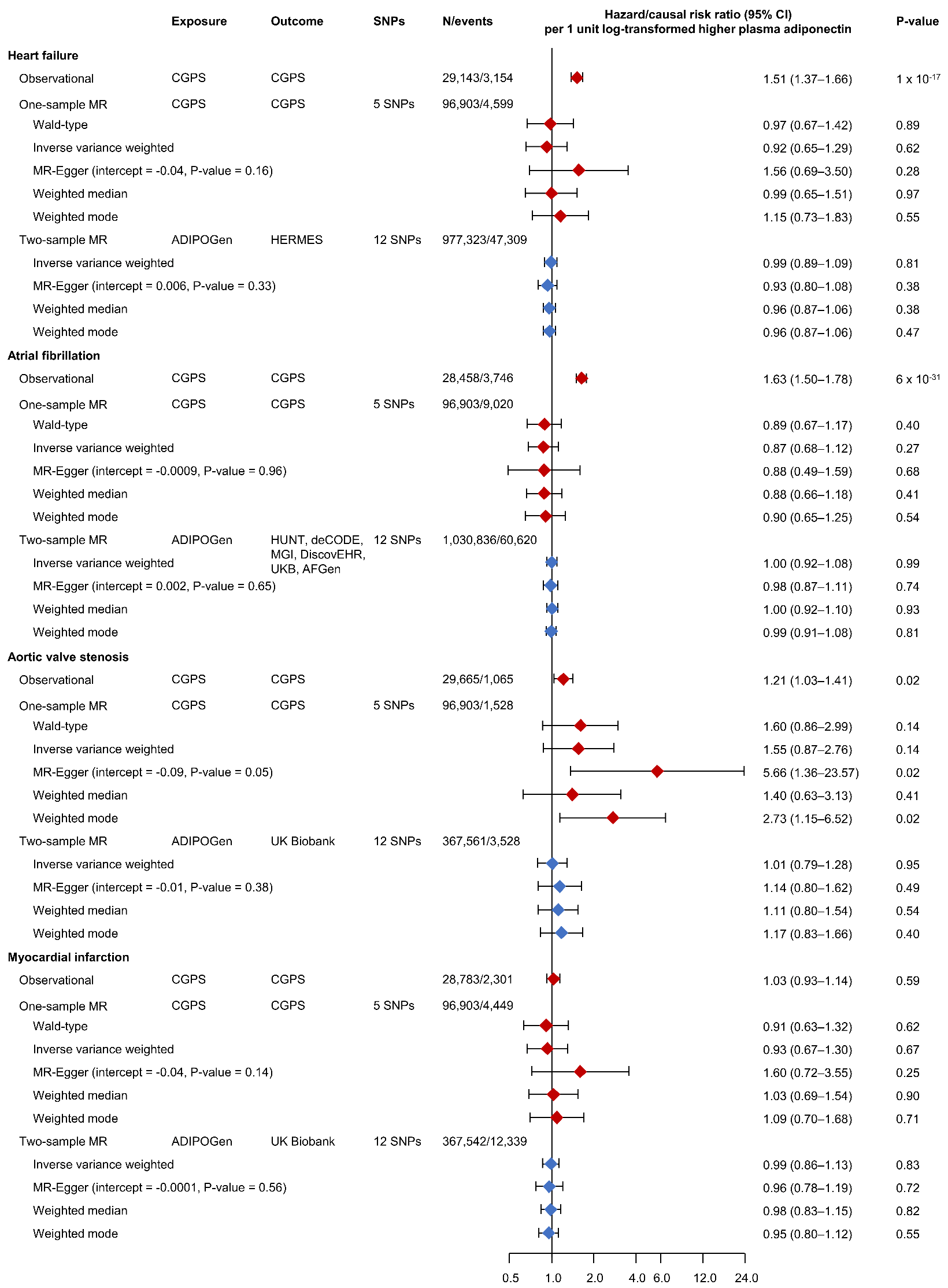
**

**Figure S13. Observational and genetic one- and two sample Mendelian association of plasma adiponectin with heart failure, atrial fibrillation, aortic valve stenosis, and myocardial infarction.** Observational analyses in the Copenhagen General Population Study (CGPS) were multivariable adjusted for age, sex, hypertension, diabetes, use of lipid-lowering drugs, smoking status, socioeconomic status, physical activity, body mass index, waist circumference, non-high-density lipoprotein cholesterol, and plasma high-sensitive C-reactive protein. The causal risk ratio with 95% confidence interval (CI) from one-sample Mendelian randomization (MR) analyses indicated with red diamonds and whiskers was based on the Copenhagen General Population Study (CGPS). The causal risk ratio with 95% confidence interval (CI) from two-sample Mendelian randomization analyses indicated with blue diamonds and whiskers was based on HERMES in heart failure; HUNT, deCODE, MGI, DiscovEHR, UKB, and AFGen in atrial fibrillation; and UK Biobank in aortic valve stenosis and myocardial infarction. Genetic information on plasma adiponectin for the two-sample Mendelian randomization was obtained from ADIPOGen. SNPs=single nucleotide polymorphisms. N=number of individuals.

**Table S4. Two-sample Mendelian randomization sensitivity analyses using UK Biobank as outcome cohorts.**

| **Heart Failure** | **Exposure cohort** | **Outcome cohort** | **N/HF** | **Method** | **SNPs** | **OR** | **95% CI** | **P-value** |
| --- | --- | --- | --- | --- | --- | --- | --- | --- |
|  | ADIPOGen | UKB | 367,542/11,841 | MR Egger | 12 | 0.94 | 0.73–1.21 | 0.63 |
|  | ADIPOGen | UKB | 367,542/11,841 | Weighted median | 12 | 0.95 | 0.79–1.14 | 0.58 |
|  | ADIPOGen | UKB | 367,542/11,841 | Inverse variance weighted | 12 | 1.02 | 0.86–1.21 | 0.84 |
|  | ADIPOGen | UKB | 367,542/11,841 | Weighted mode | 12 | 0.95 | 0.81–1.13 | 0.61 |
| **Atrial fibrillation** | **Exposure cohort** | **Outcome cohort** | **N/AF** | **Method** | **SNPs** | **OR** | **95% CI** | **P-value** |
|  | ADIPOGen | UKB | 367,542/26,172 | MR Egger | 12 | 0.98 | 0.85–1.13 | 0.81 |
|  | ADIPOGen | UKB | 367,542/26,172 | Weighted median | 12 | 0.99 | 0.88–1.12 | 0.90 |
|  | ADIPOGen | UKB | 367,542/26,172 | Inverse variance weighted | 12 | 1.00 | 0.91–1.10 | 0.98 |
|  | ADIPOGen | UKB | 367,542/26,172 | Weighted mode | 12 | 0.99 | 0.88–1.11 | 0.88 |
| **Coronary artery disease** | **Exposure cohort** | **Outcome cohort** | **N/CAD** | **Method** | **SNPs** | **OR** | **95% CI** | **P-value** |
|  | ADIPOGen | UKB | 367,561/35,979 | MR Egger | 12 | 0.83 | 0.67–1.03 | 0.13 |
|  | ADIPOGen | UKB | 367,561/35,979 | Weighted median | 12 | 0.87 | 0.78–0.97 | 0.01 |
|  | ADIPOGen | UKB | 367,561/35,979 | Inverse variance weighted | 12 | 0.90 | 0.78–1.04 | 0.14 |
|  | ADIPOGen | UKB | 367,561/35,979 | Weighted mode | 12 | 0.86 | 0.78–0.95 | 0.02 |

Odds ratio (OR) and 95% confidence interval (CI) for heart failure (HF), atrial fibrillation (AF), and coronary artery disease (CAD) when using UK Biobank (UKB) as outcome cohort.

Genetic associations in the UK Biobank were estimated using logistic regression with adjustment for age, sex, and 10 genomic principal components to account for population structure. The analytic sample was defined using a previously described approach^4^: we excluded participants with genetic sex mismatch, non-European ancestries (self-report or inferred by genetics), or excess heterozygosity (>3 standard deviations from the mean), and included only one of each set of related participants (third degree relatives or closer). SNPs=single nucleotide polymorphisms. N=number of individuals.


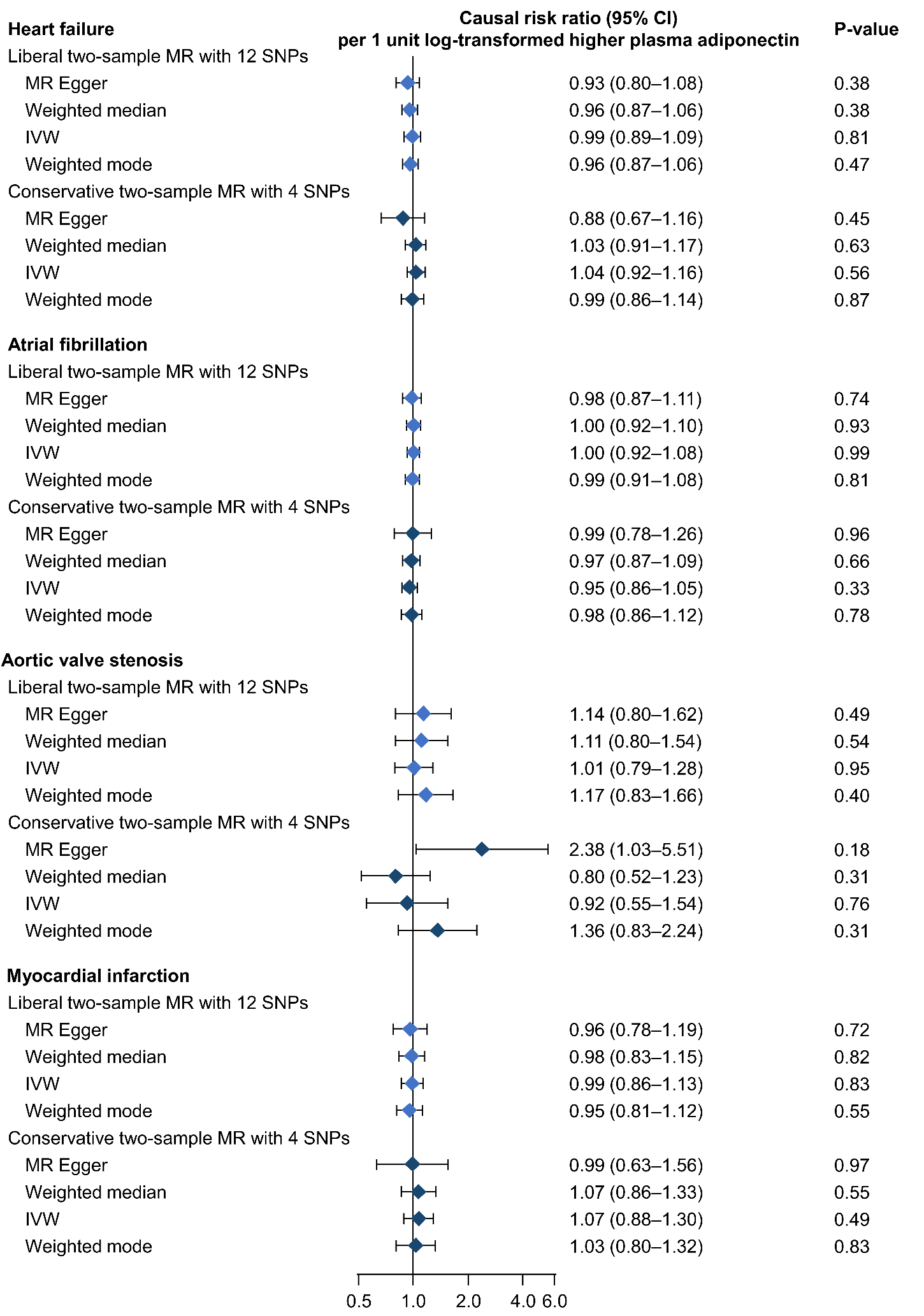


**Figure S14. Genetic two-sample Mendelian association of plasma adiponectin with heart failure, atrial fibrillation, aortic valve stenosis, and myocardial infarction using a liberal approach with all 12 SNPs and a conservative approach with 4 SNPs in the *ADIPOQ* locus.** The causal risk ratio with 95% confidence interval (CI) indicated with light and dark blue diamonds and whiskers for the liberal and conservative approach, respectively. SNPs used can be found in Table S1 blue column. Genetic information was based on HERMES in heart failure; HUNT, deCODE, MGI, DiscovEHR, UKB, and AFGen in atrial fibrillation; and UK Biobank in aortic valve stenosis and myocardial infarction. Genetic information on plasma adiponectin was obtained from ADIPOGen. SNPs=single nucleotide polymorphisms. IVW=inverse variance weighted.


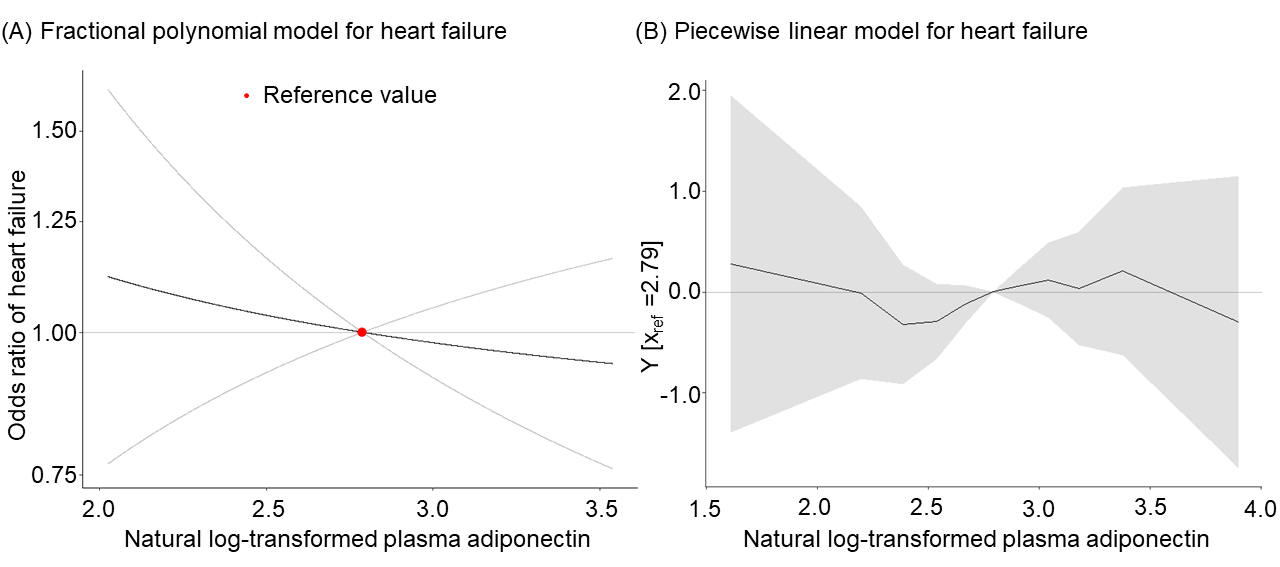


**Figure S15. Non-linear one-sample Mendelian randomization of plasma adiponectin with heart failure in the Copenhagen General Population Study.** The Copenhagen Population was divided in ten strata using the doubly-ranked stratification method to investigate non-linear effects of plasma on heart failure. Part (A) shows the fractional polynomial model and part (B) shows the piecewise linear model. The mean value of natural log-transformed plasma adiponectin in CGPS was used as the reference value. Non-linearity tests included fractional polynomial degree p-value=0.376, fractional polynomial non-linearity p-value=0.576, quadratic p-value=0.416, Cochran Q p-value=0.042. CGPS=Copenhagen General Population Study.


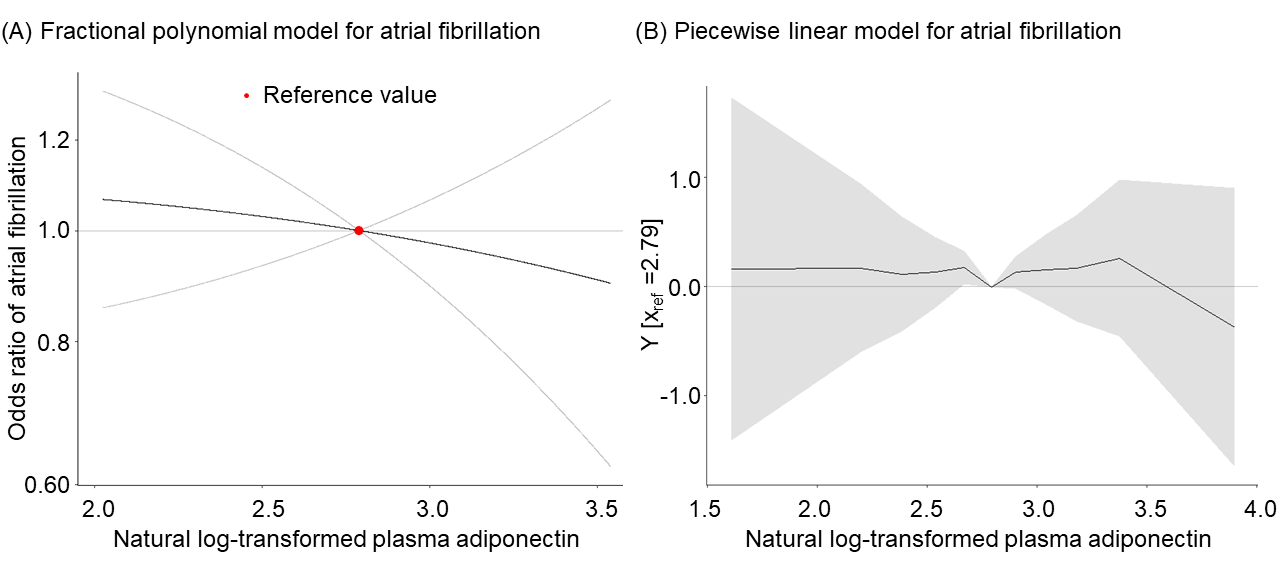


**Figure S16. Non-linear one-sample Mendelian randomization of plasma adiponectin with atrial fibrillation in the Copenhagen General Population Study.** The Copenhagen Population was divided in ten strata using the doubly-ranked stratification method to investigate non-linear effects of plasma on heart failure. Part (A) shows the fractional polynomial model and part (B) shows the piecewise linear model. The mean value of natural log-transformed plasma adiponectin in CGPS was used as the reference value. Non-linearity tests included fractional polynomial degree p-value=0.817, fractional polynomial non-linearity p-value=0.69, quadratic p-value=0.613, Cochran Q p-value=0.168. CGPS=Copenhagen General Population Study.


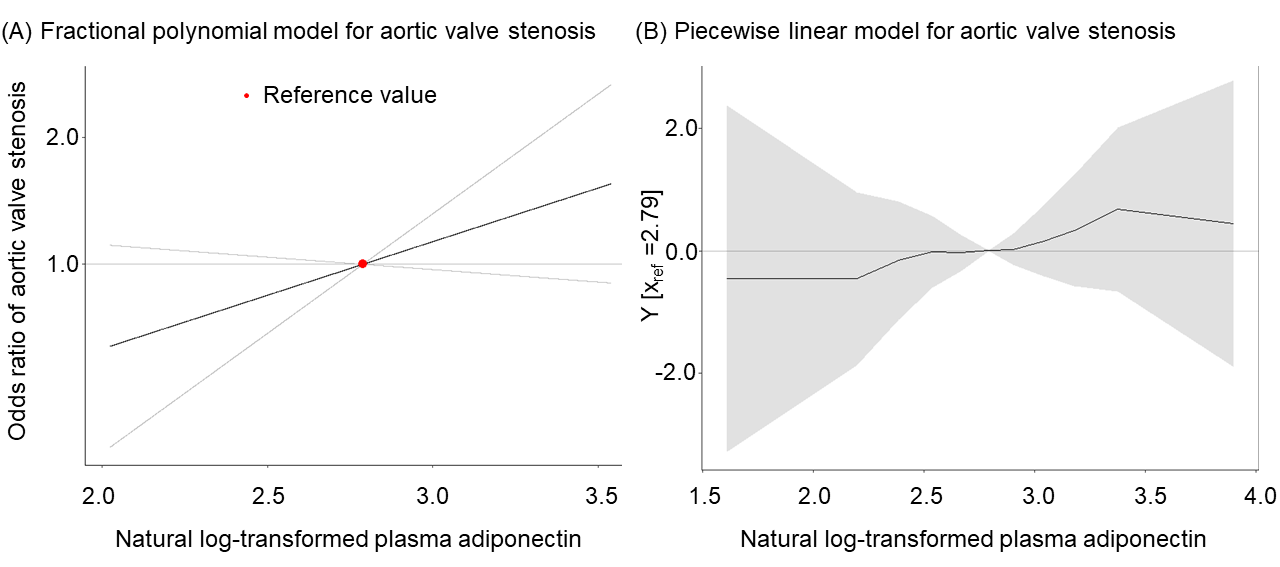


**Figure S17. Non-linear one-sample Mendelian randomization of plasma adiponectin with aortic valve stenosis in the Copenhagen General Population Study.** The Copenhagen Population was divided in ten strata using the doubly-ranked stratification method to investigate non-linear effects of plasma on heart failure. Part (A) shows the fractional polynomial model and part (B) shows the piecewise linear model. The mean value of natural log-transformed plasma adiponectin in CGPS was used as the reference value. Non-linearity tests included fractional polynomial degree p-value=0.94, fractional polynomial non-linearity p-value=1, quadratic p-value=0.99, Cochran Q p-value=0.919. CGPS=Copenhagen General Population Study.


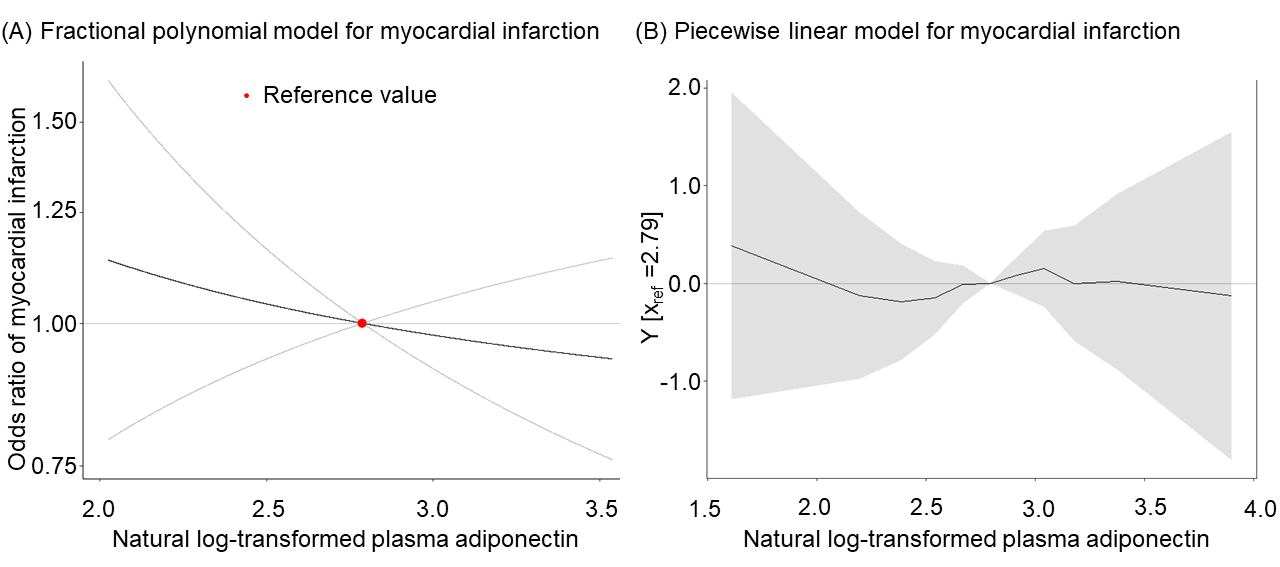


**Figure S18. Non-linear one-sample Mendelian randomization of plasma adiponectin with myocardial infarction in the Copenhagen General Population Study.** The Copenhagen Population was divided in ten strata using the doubly-ranked stratification method to investigate non-linear effects of plasma on heart failure. Part (A) shows the fractional polynomial model and part (B) shows the piecewise linear model. The mean value of natural log-transformed plasma adiponectin in CGPS was used as the reference value. Non-linearity tests included fractional polynomial degree p-value=0.344, fractional polynomial non-linearity p-value=0.525, quadratic p-value=0.362, Cochran Q p-value=0.498. CGPS=Copenhagen General Population Study.

**References**

1. Dastani Z, Hivert MF, Timpson N, Perry JR, Yuan X, Scott RA, Henneman P, Heid IM, Kizer JR, Lyytikainen LP, Fuchsberger C, Tanaka T, Morris AP, Small K, Isaacs A, Beekman M, Coassin S, Lohman K, Qi L, Kanoni S, Pankow JS, Uh HW, Wu Y, Bidulescu A, Rasmussen-Torvik LJ, Greenwood CM, Ladouceur M, Grimsby J, Manning AK, Liu CT, Kooner J, Mooser VE, Vollenweider P, Kapur KA, Chambers J, Wareham NJ, Langenberg C, Frants R, Willems-Vandijk K, Oostra BA, Willems SM, Lamina C, Winkler TW, Psaty BM, Tracy RP, Brody J, Chen I, Viikari J, Kahonen M, Pramstaller PP, Evans DM, St Pourcain B, Sattar N, Wood AR, Bandinelli S, Carlson OD, Egan JM, Bohringer S, van Heemst D, Kedenko L, Kristiansson K, Nuotio ML, Loo BM, Harris T, Garcia M, Kanaya A, Haun M, Klopp N, Wichmann HE, Deloukas P, Katsareli E, Couper DJ, Duncan BB, Kloppenburg M, Adair LS, Borja JB, Consortium D, Consortium M, Investigators G, Mu TC, Wilson JG, Musani S, Guo X, Johnson T, Semple R, Teslovich TM, Allison MA, Redline S, Buxbaum SG, Mohlke KL, Meulenbelt I, Ballantyne CM, Dedoussis GV, Hu FB, Liu Y, Paulweber B, Spector TD, Slagboom PE, Ferrucci L, Jula A, Perola M, Raitakari O, Florez JC, Salomaa V, Eriksson JG, Frayling TM, Hicks AA, Lehtimaki T, Smith GD, Siscovick DS, Kronenberg F, van Duijn C, Loos RJ, Waterworth DM, Meigs JB, Dupuis J, Richards JB, Voight BF, Scott LJ, Steinthorsdottir V, Dina C, Welch RP, Zeggini E, Huth C, Aulchenko YS, Thorleifsson G, McCulloch LJ, Ferreira T, Grallert H, Amin N, Wu G, Willer CJ, Raychaudhuri S, McCarroll SA, Hofmann OM, Segre AV, van Hoek M, Navarro P, Ardlie K, Balkau B, Benediktsson R, Bennett AJ, Blagieva R, Boerwinkle E, Bonnycastle LL, Bostrom KB, Bravenboer B, Bumpstead S, Burtt NP, Charpentier G, Chines PS, Cornelis M, Crawford G, Doney AS, Elliott KS, Elliott AL, Erdos MR, Fox CS, Franklin CS, Ganser M, Gieger C, Grarup N, Green T, Griffin S, Groves CJ, Guiducci C, Hadjadj S, Hassanali N, Herder C, Isomaa B, Jackson AU, Johnson PR, Jorgensen T, Kao WH, Kong A, Kraft P, Kuusisto J, Lauritzen T, Li M, Lieverse A, Lindgren CM, Lyssenko V, Marre M, Meitinger T, Midthjell K, Morken MA, Narisu N, Nilsson P, Owen KR, Payne F, Petersen AK, Platou C, Proenca C, Prokopenko I, Rathmann W, Rayner NW, Robertson NR, Rocheleau G, Roden M, Sampson MJ, Saxena R, Shields BM, Shrader P, Sigurdsson G, Sparso T, Strassburger K, Stringham HM, Sun Q, Swift AJ, Thorand B, Tichet J, Tuomi T, van Dam RM, van Haeften TW, van Herpt T, van Vliet-Ostaptchouk JV, Walters GB, Weedon MN, Wijmenga C, Witteman J, Bergman RN, Cauchi S, Collins FS, Gloyn AL, Gyllensten U, Hansen T, Hide WA, Hitman GA, Hofman A, Hunter DJ, Hveem K, Laakso M, Morris AD, Palmer CN, Rudan I, Sijbrands E, Stein LD, Tuomilehto J, Uitterlinden A, Walker M, Watanabe RM, Abecasis GR, Boehm BO, Campbell H, Daly MJ, Hattersley AT, Pedersen O, Barroso I, Groop L, Sladek R, Thorsteinsdottir U, Wilson JF, Illig T, Froguel P, van Duijn CM, Stefansson K, Altshuler D, Boehnke M, McCarthy MI, Soranzo N, Wheeler E, Glazer NL, Bouatia-Naji N, Magi R, Randall J, Elliott P, Rybin D, Dehghan A, Hottenga JJ, Song K, Goel A, Lajunen T, Doney A, Cavalcanti-Proenca C, Kumari M, Timpson NJ, Zabena C, Ingelsson E, An P, O'Connell J, Luan J, Elliott A, McCarroll SA, Roccasecca RM, Pattou F, Sethupathy P, Ariyurek Y, Barter P, Beilby JP, Ben-Shlomo Y, Bergmann S, Bochud M, Bonnefond A, Borch-Johnsen K, Bottcher Y, Brunner E, Bumpstead SJ, Chen YD, Chines P, Clarke R, Coin LJ, Cooper MN, Crisponi L, Day IN, de Geus EJ, Delplanque J, Fedson AC, Fischer-Rosinsky A, Forouhi NG, Franzosi MG, Galan P, Goodarzi MO, Graessler J, Grundy S, Gwilliam R, Hallmans G, Hammond N, Han X, Hartikainen AL, Hayward C, Heath SC, Hercberg S, Hillman DR, Hingorani AD, Hui J, Hung J, Kaakinen M, Kaprio J, Kesaniemi YA, Kivimaki M, Knight B, Koskinen S, Kovacs P, Kyvik KO, Lathrop GM, Lawlor DA, Le Bacquer O, Lecoeur C, Li Y, Mahley R, Mangino M, Martinez-Larrad MT, McAteer JB, McPherson R, Meisinger C, Melzer D, Meyre D, Mitchell BD, Mukherjee S, Naitza S, Neville MJ, Orru M, Pakyz R, Paolisso G, Pattaro C, Pearson D, Peden JF, Pedersen NL, Pfeiffer AF, Pichler I, Polasek O, Posthuma D, Potter SC, Pouta A, Province MA, Rayner NW, Rice K, Ripatti S, Rivadeneira F, Rolandsson O, Sandbaek A, Sandhu M, Sanna S, Sayer AA, Scheet P, Seedorf U, Sharp SJ, Shields B, Sigurethsson G, Sijbrands EJ, Silveira A, Simpson L, Singleton A, Smith NL, Sovio U, Swift A, Syddall H, Syvanen AC, Tonjes A, Uitterlinden AG, van Dijk KW, Varma D, Visvikis-Siest S, Vitart V, Vogelzangs N, Waeber G, Wagner PJ, Walley A, Ward KL, Watkins H, Wild SH, Willemsen G, Witteman JC, Yarnell JW, Zelenika D, Zethelius B, Zhai G, Zhao JH, Zillikens MC, Consortium D, Consortium G, Global BPC, Borecki IB, Meneton P, Magnusson PK, Nathan DM, Williams GH, Silander K, Bornstein SR, Schwarz P, Spranger J, Karpe F, Shuldiner AR, Cooper C, Serrano-Rios M, Lind L, Palmer LJ, Hu FBs, Franks PW, Ebrahim S, Marmot M, Kao WH, Pramstaller PP, Wright AF, Stumvoll M, Hamsten A, Procardis C, Buchanan TA, Valle TT, Rotter JI, Penninx BW, Boomsma DI, Cao A, Scuteri A, Schlessinger D, Uda M, Ruokonen A, Jarvelin MR, Peltonen L, Mooser V, Sladek R, investigators M, Consortium G, Musunuru K, Smith AV, Edmondson AC, Stylianou IM, Koseki M, Pirruccello JP, Chasman DI, Johansen CT, Fouchier SW, Peloso GM, Barbalic M, Ricketts SL, Bis JC, Feitosa MF, Orho-Melander M, Melander O, Li X, Li M, Cho YS, Go MJ, Kim YJ, Lee JY, Park T, Kim K, Sim X, Ong RT, Croteau-Chonka DC, Lange LA, Smith JD, Ziegler A, Zhang W, Zee RY, Whitfield JB, Thompson JR, Surakka I, Spector TD, Smit JH, Sinisalo J, Scott J, Saharinen J, Sabatti C, Rose LM, Roberts R, Rieder M, Parker AN, Pare G, O'Donnell CJ, Nieminen MS, Nickerson DA, Montgomery GW, McArdle W, Masson D, Martin NG, Marroni F, Lucas G, Luben R, Lokki ML, Lettre G, Launer LJ, Lakatta EG, Laaksonen R, Kyvik KO, Konig IR, Khaw KT, Kaplan LM, Johansson A, Janssens AC, Igl W, Hovingh GK, Hengstenberg C, Havulinna AS, Hastie ND, Harris TB, Haritunians T, Hall AS, Groop LC, Gonzalez E, Freimer NB, Erdmann J, Ejebe KG, Doring A, Dominiczak AF, Demissie S, Deloukas P, de Faire U, Crawford G, Chen YD, Caulfield MJ, Boekholdt SM, Assimes TL, Quertermous T, Seielstad M, Wong TY, Tai ES, Feranil AB, Kuzawa CW, Taylor HA, Jr., Gabriel SB, Holm H, Gudnason V, Krauss RM, Ordovas JM, Munroe PB, Kooner JS, Tall AR, Hegele RA, Kastelein JJ, Schadt EE, Strachan DP, Reilly MP, Samani NJ, Schunkert H, Cupples LA, Sandhu MS, Ridker PM, Rader DJ, Kathiresan S. Novel loci for adiponectin levels and their influence on type 2 diabetes and metabolic traits: a multi-ethnic meta-analysis of 45,891 individuals. *PLoS Genet* 2012;**8**:e1002607.

2. Shah S, Henry A, Roselli C, Lin H, Sveinbjornsson G, Fatemifar G, Hedman AK, Wilk JB, Morley MP, Chaffin MD, Helgadottir A, Verweij N, Dehghan A, Almgren P, Andersson C, Aragam KG, Arnlov J, Backman JD, Biggs ML, Bloom HL, Brandimarto J, Brown MR, Buckbinder L, Carey DJ, Chasman DI, Chen X, Chen X, Chung J, Chutkow W, Cook JP, Delgado GE, Denaxas S, Doney AS, Dorr M, Dudley SC, Dunn ME, Engstrom G, Esko T, Felix SB, Finan C, Ford I, Ghanbari M, Ghasemi S, Giedraitis V, Giulianini F, Gottdiener JS, Gross S, Guethbjartsson DF, Gutmann R, Haggerty CM, van der Harst P, Hyde CL, Ingelsson E, Jukema JW, Kavousi M, Khaw KT, Kleber ME, Kober L, Koekemoer A, Langenberg C, Lind L, Lindgren CM, London B, Lotta LA, Lovering RC, Luan J, Magnusson P, Mahajan A, Margulies KB, Marz W, Melander O, Mordi IR, Morgan T, Morris AD, Morris AP, Morrison AC, Nagle MW, Nelson CP, Niessner A, Niiranen T, O'Donoghue ML, Owens AT, Palmer CNA, Parry HM, Perola M, Portilla-Fernandez E, Psaty BM, Regeneron Genetics C, Rice KM, Ridker PM, Romaine SPR, Rotter JI, Salo P, Salomaa V, van Setten J, Shalaby AA, Smelser DT, Smith NL, Stender S, Stott DJ, Svensson P, Tammesoo ML, Taylor KD, Teder-Laving M, Teumer A, Thorgeirsson G, Thorsteinsdottir U, Torp-Pedersen C, Trompet S, Tyl B, Uitterlinden AG, Veluchamy A, Volker U, Voors AA, Wang X, Wareham NJ, Waterworth D, Weeke PE, Weiss R, Wiggins KL, Xing H, Yerges-Armstrong LM, Yu B, Zannad F, Zhao JH, Hemingway H, Samani NJ, McMurray JJV, Yang J, Visscher PM, Newton-Cheh C, Malarstig A, Holm H, Lubitz SA, Sattar N, Holmes MV, Cappola TP, Asselbergs FW, Hingorani AD, Kuchenbaecker K, Ellinor PT, Lang CC, Stefansson K, Smith JG, Vasan RS, Swerdlow DI, Lumbers RT. Genome-wide association and Mendelian randomisation analysis provide insights into the pathogenesis of heart failure. *Nat Commun* 2020;**11**:163.

3. Bycroft C, Freeman C, Petkova D, Band G, Elliott LT, Sharp K, Motyer A, Vukcevic D, Delaneau O, O'Connell J, Cortes A, Welsh S, Young A, Effingham M, McVean G, Leslie S, Allen N, Donnelly P, Marchini J. The UK Biobank resource with deep phenotyping and genomic data. *Nature* 2018;**562**:203-209.

4. Astle WJ, Elding H, Jiang T, Allen D, Ruklisa D, Mann AL, Mead D, Bouman H, Riveros-Mckay F, Kostadima MA, Lambourne JJ, Sivapalaratnam S, Downes K, Kundu K, Bomba L, Berentsen K, Bradley JR, Daugherty LC, Delaneau O, Freson K, Garner SF, Grassi L, Guerrero J, Haimel M, Janssen-Megens EM, Kaan A, Kamat M, Kim B, Mandoli A, Marchini J, Martens JHA, Meacham S, Megy K, O'Connell J, Petersen R, Sharifi N, Sheard SM, Staley JR, Tuna S, van der Ent M, Walter K, Wang SY, Wheeler E, Wilder SP, Iotchkova V, Moore C, Sambrook J, Stunnenberg HG, Di Angelantonio E, Kaptoge S, Kuijpers TW, Carrillo-de-Santa-Pau E, Juan D, Rico D, Valencia A, Chen L, Ge B, Vasquez L, Kwan T, Garrido-Martin D, Watt S, Yang Y, Guigo R, Beck S, Paul DS, Pastinen T, Bujold D, Bourque G, Frontini M, Danesh J, Roberts DJ, Ouwehand WH, Butterworth AS, Soranzo N. The Allelic Landscape of Human Blood Cell Trait Variation and Links to Common Complex Disease. *Cell* 2016;**167**:1415-1429 e1419.

5. Nielsen JB, Thorolfsdottir RB, Fritsche LG, Zhou W, Skov MW, Graham SE, Herron TJ, McCarthy S, Schmidt EM, Sveinbjornsson G, Surakka I, Mathis MR, Yamazaki M, Crawford RD, Gabrielsen ME, Skogholt AH, Holmen OL, Lin M, Wolford BN, Dey R, Dalen H, Sulem P, Chung JH, Backman JD, Arnar DO, Thorsteinsdottir U, Baras A, O'Dushlaine C, Holst AG, Wen X, Hornsby W, Dewey FE, Boehnke M, Kheterpal S, Mukherjee B, Lee S, Kang HM, Holm H, Kitzman J, Shavit JA, Jalife J, Brummett CM, Teslovich TM, Carey DJ, Gudbjartsson DF, Stefansson K, Abecasis GR, Hveem K, Willer CJ. Biobank-driven genomic discovery yields new insight into atrial fibrillation biology. *Nat Genet* 2018;**50**:1234-1239.

6. Kamat MA, Blackshaw JA, Young R, Surendran P, Burgess S, Danesh J, Butterworth AS, Staley JR. PhenoScanner V2: an expanded tool for searching human genotype-phenotype associations. *Bioinformatics* 2019;**35**:4851-4853.

7. Staley JR, Blackshaw J, Kamat MA, Ellis S, Surendran P, Sun BB, Paul DS, Freitag D, Burgess S, Danesh J, Young R, Butterworth AS. PhenoScanner: a database of human genotype-phenotype associations. *Bioinformatics* 2016;**32**:3207-3209.

8. Machiela MJ, Chanock SJ. LDlink: a web-based application for exploring population-specific haplotype structure and linking correlated alleles of possible functional variants. *Bioinformatics* 2015;**31**:3555-3557.

9. Burgess S, Davey Smith G, Davies NM, Dudbridge F, Gill D, Glymour MM, Hartwig FP, Holmes MV, Minelli C, Relton CL, Theodoratou E. Guidelines for performing Mendelian randomization investigations. *Wellcome Open Res* 2019;**4**:186.
